# A Non‐Mitophagy Activity of BNIP3L/NIX in Amygdala Glutamatergic Neurons is Essential for Contextual Fear Memory Formation

**DOI:** 10.1002/advs.202517585

**Published:** 2026-01-25

**Authors:** Xingxian Zhang, Xinlei Mo, Xinyu Zhou, Xiaoliang Liu, Guizhi Li, Songhui Hu, Yangyang Lu, Chenze Zhu, Jinxi Feng, Zhitong Chen, Weiwei Hu, Yihui Cui, Zhong Chen, Xiangnan Zhang

**Affiliations:** ^1^ Institute of Pharmacology & Toxicology Zhejiang Key Laboratory of Neuropsychopharmacology State Key Laboratory of Advanced Drug Delivery and Release Systems College of Pharmaceutical Sciences Zhejiang University Hangzhou China; ^2^ School of Pharmacy Hangzhou Medical College Hangzhou Zhejiang China; ^3^ Department of Pharmacology and Department of Pharmacy of the Second Affiliated Hospital School of Basic Medical Sciences Zhejiang University Hangzhou Zhejiang China; ^4^ Jinhua Institute of Zhejiang University Jinhua Zhejiang China; ^5^ Department of Neurobiology Department of Neurology of Sir Run Run Shaw Hospital Zhejiang University School of Medicine Hangzhou Zhejiang China; ^6^ Key Laboratory of Neuropharmacology and Translational Medicine of Zhejiang Province College of Pharmaceutical Sciences Zhejiang Chinese Medical University Hangzhou Zhejiang China

**Keywords:** AMPK‐Drp1, basolateral amygdala, BNIP3L/NIX, contextual fear memory, mitochondrial fission

## Abstract

Mitochondrial quality is crucial for maintaining brain homeostasis. BNIP3L/NIX, a mitophagy receptor, has been linked to neurological disorders, yet its specific function in the brain remains unclear. We found BNIP3L highly expressed in basolateral amygdala (BLA) neurons. Selective deletion of *bnip3l* in BLA glutamatergic neurons (BLA^GLU^) impaired contextual fear memory, accompanied by reduced neuronal excitation and mitochondrial respiration. Notably, fear conditioning did not invariably activate mitophagy in BLA^GLU^ neurons. Overexpression of both wild‐type and a mitophagy‐deficient mutant (BNIP3L^ΔLIR^) in BLA^GLU^ neurons was sufficient to rescue the contextual fear memory deficits in *bnip3l* ^−/−^ mice, suggesting a non‐mitophagy role. Instead, we detected a prompt mitochondrial fission in BLA^GLU^ neurons after foot‐shock conditioning, an effect abolished by *bnip3l* deletion. Inhibition of Drp1 with Mdivi‐1 disrupted memory formation, whereas optogenetic activation of Drp1 restored neuronal excitation and rescued memory deficits in *bnip3l* ^−/−^ mice. These data indicated an essential role of BNIP3L‐mediated mitochondrial fission in modulating contextual fear memory. Mechanistically, BNIP3L and Drp1 competitively interact with AMPK, leading to reduced Drp1 phosphorylation and increased Drp1 accumulation on mitochondria, thereby promoting mitochondrial fission. Taken together, the present study revealed a previously uncharacterized, non‐mitophagy‐dependent role for BNIP3L in contextual fear memory conditioning.

## Graphical abstract

Contextual fear conditioning induces BNIP3L‐dependent mitochondrial fission in glutamatergic neurons of the BLA, independently of mitophagy. Loss of BNIP3L elevates Drp1^Ser637^ phosphorylation, thereby suppressing mitochondrial fission, compromising ATP production, and attenuating excitatory synaptic transmission. These bioenergetic and synaptic deficits ultimately impair contextual fear memory formation, revealing a critical non‐mitophagic role of BNIP3L underlying emotional memory.

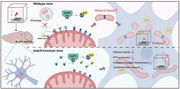

**Abbreviations**
AAV: adeno‐associated virus
AHP: after‐hyperpolarization
AMPK: AMP‐activated protein kinase
ATG7: autophagy related protein 7
ATP: Adenosine Triphosphate
BLA: basolateral amygdala
BNIP3L/NIX: BCL2/adenovirus E1B interacting protein 3‐like
CaMKIIɑ: Calcium/Calmodulin‐Dependent Protein Kinase II Alpha
CeA: central amygdala
CIB1: cryptochrome‐interacting basic‐helix‐loop‐helix 1
Cry2: cryptochrome 2
DIO: Double‐floxed inverse orientation
Drp1: Dynamin‐related protein 1
FC: fear‐conditioning
GFP: green fluorescent protein
HC: home‐cage
Hsp60: heat shock protein 60
LC3: microtube‐associated protein 1 light chain 3
LIR: LC3‐interacting region
LTP: long‐term potentiation
Mdivi‐1: mitochondrial fission inhibitor 1
mPFC: medial prefrontal cortex
OCR: oxygen consumption rate
PARK2: Parkinson disease (autosomal recessive juvenile) 2, parkin
PLA: proximity ligation assay
PVT: paraventricular thalamic nucleus
sEPSC: spontaneous excitatory postsynaptic currents
SQSTM1/p62: sequestosome 1
TOMM20: translocase of outer mitochondrial membrane 20 homolog (yeast)
Tr: trail
vCA1: ventral CA1 region of the hippocampus
WT: Wild‐type

## 1 Introduction

Mitochondria are crucial organelles in maintaining brain homeostasis. Neuronal mitochondria undergo a dynamic change of shapes to orchestrate with neuronal activity [1]. Divided mitochondria are distributed to fine neuronal compartments like synapses to meet the needs of energy and synaptic transmission [2, 3, 4, 5, 6], while the mitochondrial fusion enhances functional stability and resilience to stress‐induced disturbances [7, 8, 9]. Dysregulation of mitochondrial dynamics has been implicated in various neurological disorders, including Parkinson's disease, Alzheimer's disease, and psychiatric disease.

Neurons have evolved various mechanisms to monitor mitochondrial quality through either the generation of new mitochondria or, more importantly, the elimination of targeted ones via mitophagy [10]. This quality control process is regulated through two distinct mechanisms: ubiquitin‐dependent and mitophagy receptor‐dependent pathways. The PINK1/Parkin pathway senses mitochondrial depolarization and facilitates the ubiquitination of mitochondrial proteins to amplify mitophagy signaling [11]. Paradoxically, PINK1 and Parkin have minimal impact on neuronal function in physiological status [12]. Furthermore, although multiple mitophagy receptors are abundantly expressed in the brain [13, 14, 15], their specific neurological functions in physiological conditions remain largely enigmatic beyond the hypothesis that redundant mitophagy activity is preserved for mitochondrial surveillance in response to stressful conditions.

BNIP3L/NIX is a mitophagy receptor that binds with ATG8 family proteins on autophagosomes to engulf target mitochondrial for degradation [16]. *Bnip3l* knockout in mice impedes the development of reticulocyte and retinal ganglia cells due to insufficient removal of mitochondria [17, 18]. In brains, BNIP3L‐mediated mitophagy is essential to prevent ischemic brain injury by eliminating damaged mitochondria [19], and BNIP3L deficiency leads to neuroinflammation and consequently depression‐like behavior in mice [20]. Intriguingly, BNIP3L is abundantly expressed in the developing brains [15], and its transcriptional variations are related to human schizophrenia [21]. Besides mitophagy induction, ectopically BNIP3L overexpression promotes mitochondrial fragmentation [22, 23], which is likely a prerequisite for mitophagy [24]. However, a direct molecular link between BNIP3L and mitochondrial dynamic changes has been missing. The physiological roles of BNIP3L in neurons, particularly regarding its influence on neuronal activity, remain poorly defined.

The present study identified a previously uncharacterized function of BNIP3L in regulating mitochondrial dynamics during fear memory formation, beyond its established role in mitophagy. We found that BNIP3L is expressed in glutamatergic neurons of the basolateral amygdala (BLA^GLU^), a key region for contextual fear memory. Notably, BNIP3L‐mediated mitochondrial fission, rather than mitophagy, proved essential for appropriate contextual fear memory conditioning. These findings highlight a novel role for BNIP3L in modulating neuronal mitochondrial dynamics and reveal a previously unknown mechanism by which mitochondrial remodeling influences emotional memory processing.

## 2 Results

### 2.1 *Bnip3l^−/−^* Mice Exhibit Impaired Contextual Fear Memory Formation

**FIGURE 1:**
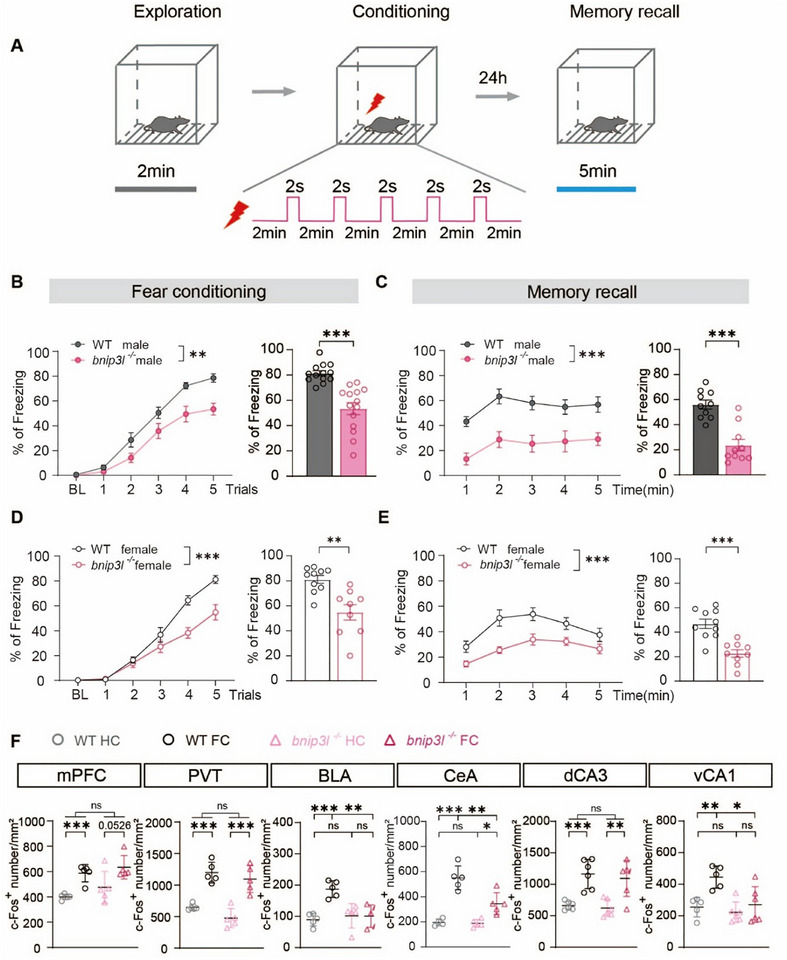
BNIP3L deletion impairs contextual fear memory formation. (A) Schematic diagram of the contextual fear memory. (B,D) The curve of freezing level (left) and the percentage of freezing time (right) on the contextual fear conditioning day. (C,E) The curve of freezing level (left) and the percentage of freezing time (right) on the contextual fear recall day (*n* = 12–14 male mice per group, *n* = 9–10 female mice per group). (F) Quantitative analysis of c‐Fos^+^ neurons number (*n* = 5 mice from per group). mPFC, medial frontal polar cortex; PVT, paraventricular nucleus of the thalamus; BLA, basolateral amygdala nucleus; CeA, central amygdala nucleus; dCA3, dorsal CA3 region of hippocampus; vCA1, ventral CA1 region of hippocampus. Data are expressed as mean ± SEM. Statistical comparisons were performed as follows: two‐way repeated measures ANOVA for B‐E (left), and G; unpaired *t*‐test for B‐E (right). ^*^ *p* < 0.05; ^**^ *p* < 0.01; ^***^ *p* < 0.001; n.s. vs. the indicated group.

To analyze the physiologically relevant functions of *bnip3l* in the brain, we determined the BNIP3L expression in the adult mouse brain by immunostaining (Figure S1A). A detailed analysis revealed brain regions with relatively high BNIP3L abundance, including the medial prefrontal cortex (mPFC), paraventricular nucleus of the thalamus (PVT), basolateral amygdala (BLA), central amygdala (CeA), and ventral CA1 region of the hippocampus(vCA1) (Figure S1B,C). Given that these BNIP3L‐enriched brain regions are critical for encoding fear and negative emotional memories [25, 26, 27], we subjected *bnip3l^−/−^* mice and their wild‐type (WT) littermates to canonical contextual fear conditioning (Figure 1A). Animals were trained to associate a neutral environment with an aversive foot‐shock, and memory formation was assayed by measuring freezing behavior. Interestingly, male *bnip3l^−/−^* mice exhibited a significantly lower freezing response (Figure 1B). Twenty‐four hours after the training trials, the *bnip3l^−/−^* mice demonstrated a reduced freezing behavior with WT controls (Figure 1C), indicating impaired aversive memory recall [28]. Since sex differences in fear memory exist [29, 30, 31, 32], female mice were also examined. The deficits in contextual fear memory were also observed in female *bnip3l^−/−^* mice (Figure 1D,E), suggesting a sex‐independent mechanism of BNIP3L in modulating fear memory formation. We did not observe any significant alterations in body growth rate, body temperature, pain sensitivity, repetitive behavior, and prepulse inhibition in *bnip3l^−/−^* mice compared with WT mice (Figure S2A—F). *Bnip3l^−/−^* mice exhibited similar motor coordination in the rotarod test and locomotor activity in open field tests as WT mice (Figure S2G,H). Additionally, *bnip3l^−/−^* mice showed no change of anxiety‐like behaviors, including time spent in the central area of the open‐field and preference for staying in the light chamber in the light‐dark box test (Figure S3A,B). *Bnip3l^−/−^* mice did not exhibit depression‐like behaviors during sucrose preference, forced swimming, and tail suspension tests (Figure S3C–E). These findings suggest that the impaired contextual fear memory formation in *bnip3l^−/−^* mice was unlikely due to differences in their pain sensitivity, acoustic response, and aversive emotion. Furthermore, *bnip3l^−/−^* mice performed normally in the three‐chamber test, spatial working memory (Y maze), novel object recognition test, and spatial reference memory (Morris water maze). Moreover, since BLA is key to encoding cued fear memory, we also examined this behavior in *bnip3l^−/−^* mice and found no significant differences (Figure S4). These data indicated that *bnip3l* knockout selectively impaired contextual fear memory, but not social behavior or other types of memory.

### 2.2 *BNIP3L* in *BLA^G^ ^LU^* Neurons is Essential for Contextual Fear Memory Formation

We next explored the brain regions that potentially contribute to the observed conditioned fear memory impairment in *bnip3l^−/−^* mice. To identify key regions involved, we analyzed neuronal activation in BNIP3L‐enriched brain regions following fear conditioning by c‐Fos immunostaining (Figure S5). Immediately following a 5‐trail foot‐shock, WT mice exhibited significantly increased c‐Fos^+^ neurons across brain regions, including mPFC, PVT, BLA, CeA, dCA3 and vCA1. Notably, *bnip3l^−/−^* mice failed to show a corresponding increase of c‐Fos^+^ neurons in BLA and vCA1 after fear conditioning (Figure 1F). These findings suggested that neuronal BNIP3L in BLA and vCA1 may play a critical role in conditioned fear memory.

**FIGURE 2:**
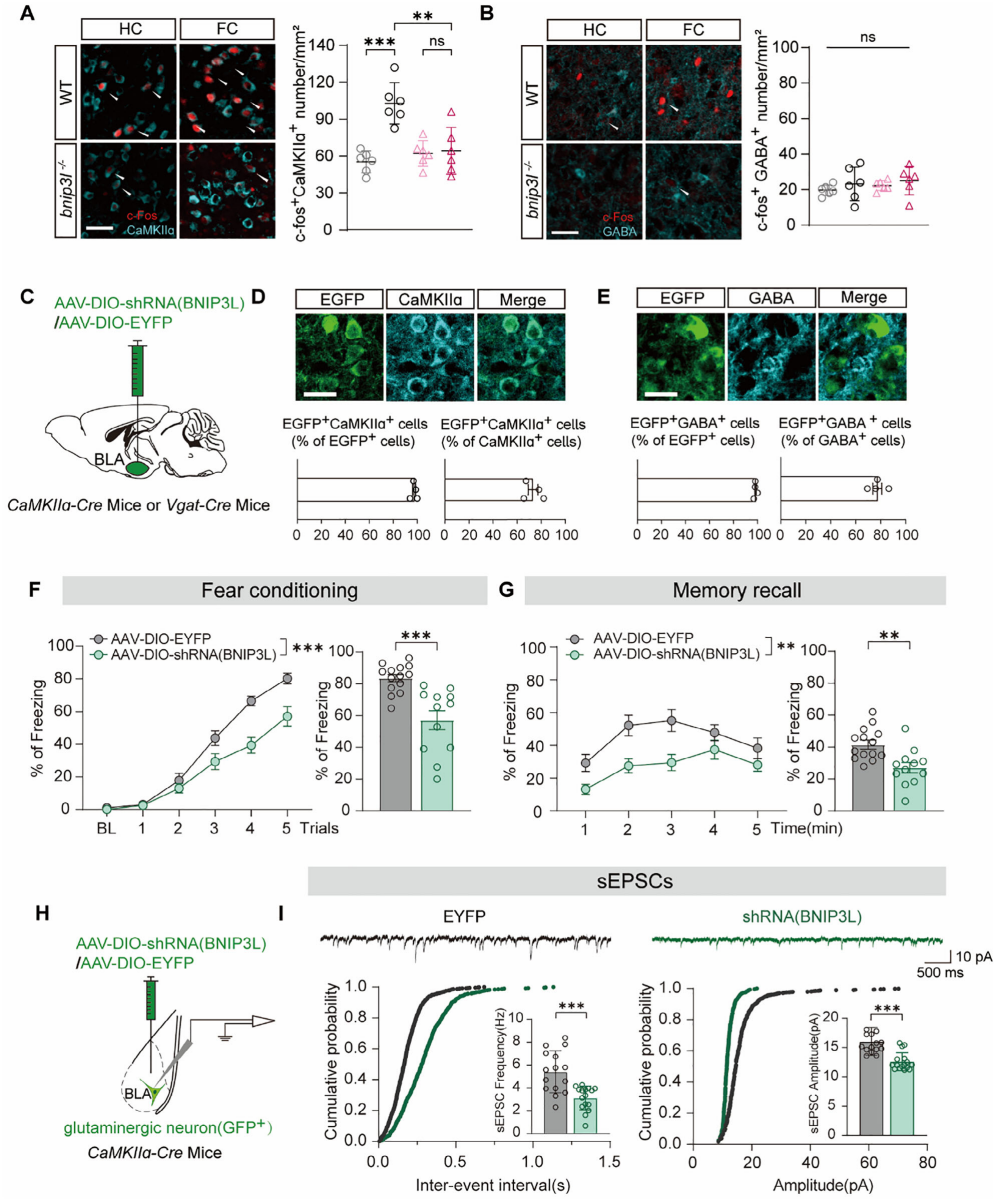
BNIP3L in the BLA glutamatergic neurons is critical for contextual fear memory conditioning. (A) Representative images of c‐Fos^+^ neurons together with CaMKIIɑ^+^ neurons in BLA after fear conditioning (left) and the number of c‐Fos^+^ neurons per ROI in the BLA (right). The neuron indicated by the white arrow in the figure shows positive expression of both c‐fos and CaMKIIɑ. Scale bar = 40 µm. *n* = 6 mice per group. (B) Representative images of c‐Fos^+^ neurons together with GABA^+^ neurons in the BLA after fear conditioning (left) and the number of c‐Fos^+^ neurons per ROI in the BLA (right). The neuron indicated by the white arrow in the figure shows positive expression of both c‐fos and GAD. Scale bar = 40 µm. *n* = 6 mice per group. (C) Experiment diagram for virus injection. *CaMKIIɑ*‐Cre or *Vgat*‐Cre mice were injected with AAV‐DIO‐EYFP or AAV‐DIO‐shRNA (BNIP3L)‐EGFP in the BLA. (D) Representative images of GFP (green), CaMKIIɑ (cyan) expression in BLA of mice after the microinjection of AAV (top). The percentage of CaMKIIɑ and EGFP co‐expressing neurons (EGFP^+^ CaMKIIɑ*^+^*) in EGFP^+^ neurons and glutamatergic neurons is shown in the columns(bottom). Scale bar = 15 µm. *n* = 3 mice per group. (E) Representative images of GFP (green), GABA (cyan) expression in the BLA of mice after the microinjection of AAV (top). The percentage of GABA and EGFP co‐expressing neurons (EGFP^+^ GABA*^+^*) in EGFP^+^ neurons and GABAergic neurons is shown in the columns (bottom). Scale bar = 15 µm. *n* = 3 mice per group. (F) The curve of freezing level (left) and the percentage of freezing time (right) on the contextual fear conditioning day. *n* = 12–14 mice per group. (G) The curve of freezing level (left) and the percentage of freezing time (right) on the contextual fear recall day. n = 12–14 mice per group. (H) Schematic diagram of BLA glutamatergic neuron recorded by whole‐cell‐patch‐clamp. (I) Representative trace of sEPSC (top), cumulative plot of sEPSC interevent intervals (bottom left) with sEPSC frequency (insert left), cumulative plot of sEPSC interevent intervals (bottom right) with sEPSC amplitude (insert right) *n* = 15 cells from 5 Ctrl mice. *n* = 16 cells from 5 BLA^GLU^ ‐shRNA (BNIP3L) mice. Statistical comparisons were performed as follows: two‐way repeated measures ANOVA for A‐B, F‐G (left), and unpaired *t*‐test for F‐G (right) and I‐J. ^*^p < 0.05; ^**^p < 0.01; ^***^p < 0.001; n.s. vs. the indicated group.

We silenced BNIP3L in vCA1^GLU^ neurons by injecting adeno‐associated virus (AAV)‐DIO‐shRNA (BNIP3L)‐EYFP into *CaMKIIɑ‐Cre* mice (vCA1^GLU^‐shBNIP3L), which led to unchanged contextual fear memory formation (Figure S6). We next determined the functional significance of BNIP3L in BLA, which is composed of approximately 80% glutamatergic neurons and 20% GABAergic neurons [33, 34]. Further analysis of c‐Fos^+^ neurons in BLA revealed that glutamatergic, rather than GABAergic neurons, were activated by fear conditioning, which was abolished in *bnip3l^−/−^* mice (Figure 2A,B). We thus selectively silenced BNIP3L in BLA neurons by bilaterally injecting AAV‐DIO‐shRNA (BNIP3L)‐GFP into BLA of *CaMKIIɑ‐Cre* mice (BLA^GLU^‐shBNIP3L) or *Vgat‐Cre* mice (BLA^GABA^‐shBNIP3L). The control mice received AAV‐DIO‐EYFP injection into the BLA (Figure 2C). The efficiency and neuron‐type specificity of viral transfection were confirmed (Figure 2D,E). After a 3‐week recovery of virus injection, mice were subjected to contextual fear conditioning. In line with the observation in *bnip3l^−/−^* mice, BLA^GLU^‐shBNIP3L mice also exhibited impaired contextual fear memory (Figure 2F,G), without affecting their locomotor activity, pain sensitivity, and cued fear memory (Figure S7). As a comparison, the BLA^GABA^‐shBNIP3L mice showed intact fear memory conditioning (Figure S8).

BLA^GLU^ neurons fire in response to fear conditioning [35, 36]. By determining spontaneous excitatory postsynaptic currents (sEPSC) in virally transfected neurons (GFP^+^), we found both the frequency and amplitude of sEPSC were significantly reduced in BLA^GLU^‐shBNIP3L neurons, compared with those from control neurons (Figure 2H,I), with their action potential threshold, amplitude, after‐hyperpolarization (AHP) amplitude, AHP time and the firing frequency unchanged (Figure S9). These results established a physiological role of BNIP3L in regulating excitatory synaptic input to BLA^GLU^ neurons. Together, these data strongly suggest that BNIP3L in BLA^GLU^ neuron is critical for contextual fear memory formation.

### 2.3 Mitophagy Activity Is Not Required for *BNIP3L* in Contextual Fear Memory Conditioning

**FIGURE 3:**
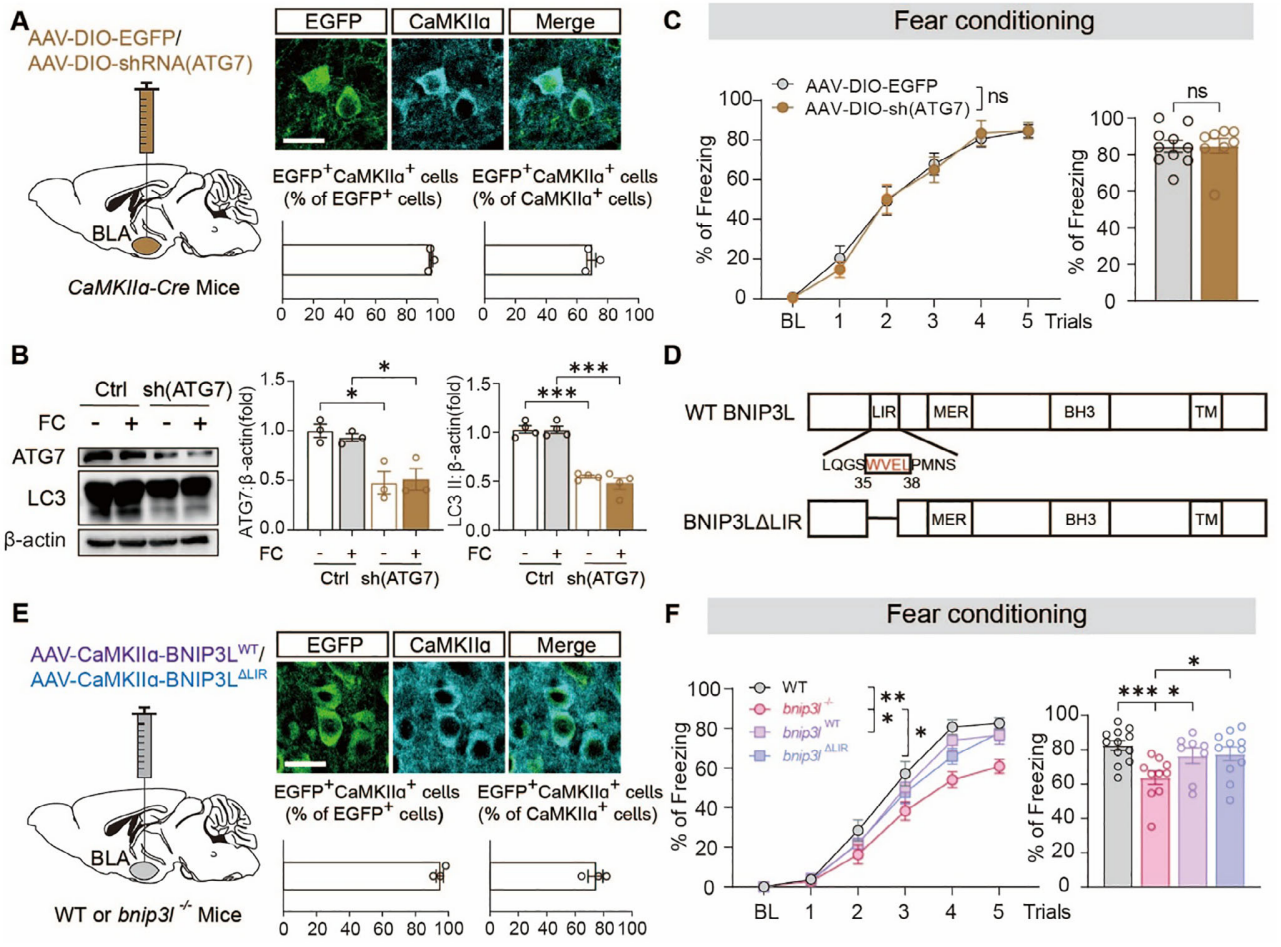
BNIP3L mediates contextual fear memory independently of its mitophagy activity. (A) Schematic diagram of virus injection. *CaMKIIɑ‐Cre* mice were injected with AAV‐DIO‐EGFP or AAV‐DIO‐shRNA (ATG7)‐EGFP in the BLA (left). Representative images of GFP (green) and CaMKIIɑ (cyan) expression in the BLA of *CaMKIIɑ‐Cre* mice after the microinjection of AAV (right, top). The percentage of CaMKIIɑ and EGFP co‐expressing neurons (EGFP^+^ *CaMKIIɑ^+^*) in EGFP^+^ neurons and glutamatergic neurons is shown in the columns (right, bottom). Scale bar = 15 µm. *n* = 3 mice per group. (B) The level of ATG7 and LC3 protein following AAV‐shRNA (ATG7/control) injection into the BLA of *CaMKIIɑ‐Cre* mice. *n* = 6 mice per group. (C) The curve of freezing level (left) and the percentage of freezing time (right) on the contextual fear conditioning day. *n* = 9–10 mice per group. (D) A scheme of BNIP3L domain organization. (E) WT or *bnip3l^−/−^* mice were injected with AAV‐CaMKIIɑ‐BNIP3L^WT^ or AAV‐CaMKIIɑ‐BNIP3L^ΔLIR^ in the BLA. Representative images of GFP (green) and CaMKIIɑ (cyan) expression in BLA of *CaMKIIɑ‐Cre* mice after the microinjection of AAV (right, top). The percentage of CaMKIIɑ and EGFP co‐expressing neurons (EGFP^+^ *CaMKIIɑ^+^*) in EGFP^+^ neurons and glutamatergic neurons is shown in the columns (right, bottom). Scale bar = 15 µm. *n* = 3 mice per group. (F) The curve of freezing level in each trial (left) and the percentage of freezing time during the contextual fear conditioning (right). *n* = 8–12 mice per group. Statistical comparisons were performed as follows: two‐way repeated measures ANOVA for B, C (left), F, and unpaired *t*‐test for C (right). ^*^p < 0.05; ^**^p < 0.01; ^***^p < 0.001; n.s. vs. the indicated group.

Increasing evidence supports a role of mitophagy in regulating synaptic homeostasis by controlling neuronal mitochondrial quality and quantity [37, 38, 39]. As a mitophagy receptor, BNIP3L monitors neuronal mitochondria in response to stress conditions [20, 40, 41]. We therefore hypothesized an essential role of BNIP3L‐mediated mitophagy in contextual fear memory formation. We detected the mitophagy flux in the BLA region with increasing trails of foot‐shock. However, foot‐shock accumulation did not alter the SQSTM1 and LC3 expression, nor did it impact mitochondrial marker TOMM20 in the BLA tissues, leaving the CCCP induced remarkable increasing of LC3 and decrease in SQSTM1 and TOMM20 (Figure S10). We further silenced *atg7* virally to inhibit autophagy in BLA^GLU^ neurons. The efficiency and specificity of viral transfection were confirmed (Figure 3A,B). Unexpectedly, it showed that *atg7* silencing in the BLA neurons had little impact on the contextual fear memory formation (Figure 3C), indicating a non‐essential role of autophagy.

BNIP3L binds to ATG8 family proteins via a conserved LC3‐interacting region (LIR) motif (Figure 3D) [16, 42, 43, 44, 45]. We determined the mitophagy flux before and after the foot‐shock in BLA^GLU^‐shBNIP3L and WT mice. Foot‐shock did not change the protein levels of SQSTM1, LC3, and TOMM20, indicating that foot‐shock did not alter mitophagy in these mice regardless of their genotypes (Figure S11). Either LIR‐deleted BNIP3L (BNIP3L^ΔLIR^) or WT BNIP3L (BNIP3L^WT^) was ectopically expressed in *bnip3l^−/−^* mice BLA^GLU^ neurons via AAVs, resulting in approximately 74.3% of CaMKIIɑ‐positive neurons infected (Figure 3E). Remarkably, both BNIP3L^WT^ and BNIP3L^ΔLIR^ overexpression in BLA^GLU^ neurons were competent to rescue the impaired fear memory conditioning in *bnip3l^−/−^* mice (Figure 3F). Overall, these data demonstrated that mitophagy activity is not required for BNIP3L in contextual fear memory.

### 2.4 Ablation of *Bnip3l* Impairs Mitochondrial Fission in *BLA^G^ ^LU^* Neurons following Fear Conditioning

**FIGURE 4:**
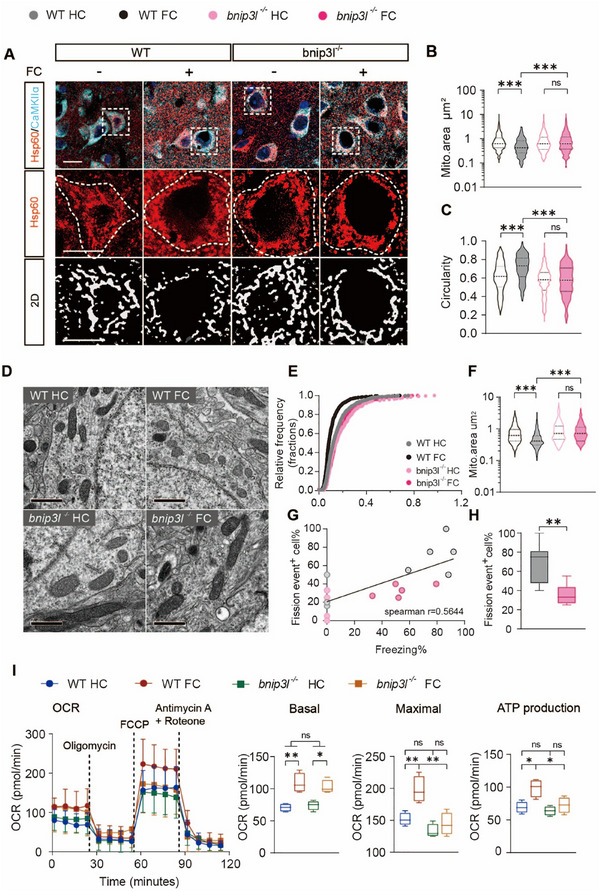
BNIP3L deletion impairs foot‐shock‐induced mitochondrial fission and mitochondrial respiration in the BLA neurons. (A) Representative images of mitochondria marker Hsp60 together with CaMKIIɑ^+^ neurons in BLA of WT or *bnip3l^−/−^* mice after fear conditioning. Scale bar = 15 µm (top). Scale bar = 10 µm (middle, bottom). *n* = 5 mice per group. (B,C) Mitochondrial morphology analysis of neurons by mean area (B) and circularity (C) per mitochondria. *n* = 6 mice per group. (D–H) Mitochondrial morphology analysis of neurons by the mean area of each mitochondrion. (D) Representative TEM images of the BLA neuron from WT or *bnip3l^−/−^* mice. Analysis of mitochondria area (E,F) and fission events of each neuron (G,H). Scale bar = 1 µm. *n* = 6–8 mice per group. (I) Mitochondrial oxygen consumption, basal respiration, maximal respiration, ATP production, and respiratory reserve capacity. *n* = 4 mice per group. Statistical comparisons were performed as follows: two‐way repeated measures ANOVA for B, C, F, and I, unpaired *t*‐test for H. ^*^p < 0.05; ^**^p < 0.01; ^***^p < 0.001; n.s. vs. the indicated group.

Mitochondria are dynamically regulated to meet the evolving needs of neurons [46]. We visualized neuronal mitochondria in the BLA before and after foot‐shock. Neuronal mitochondria were analyzed in the BLA of WT and *bnip3l^−/−^* mice immediately experienced contextual fear conditioning. By immunostaining mitochondria (Hsp60) in glutamatergic (CaMKIIɑ^+^) neurons, we observed a significant mitochondrial fragmentation in the BLA following foot‐shock, as evidenced by a remarkably decreased mitochondrial area and increased mitochondrial circularity in WT mice. Notably, these morphological changes were absent in *bnip3l^−/−^* mice (Figure 4A–C). These unexpected alterations of mitochondria dynamics in BLA neurons were confirmed by transmission electron microscopy (TEM), which also revealed an increased population of fragmented mitochondria upon a 5‐trial foot‐shock in WT but not *bnip3l^−/−^* mice (Figure 4D–F). Additionally, a positive correlation was demonstrated between the duration of freezing behavior in each mouse and the occurrence of mitochondrial fission events in its BLA neurons (Figure 4G,H). Mitochondria serve as the principal source of neuronal energy [47]. Subsequently, mitochondrial respiratory function of the BLA was assessed by Seahorse XF. Compared to the home‐cage (HC) group, the fear‐conditioned (FC) WT mice exhibited improved mitochondrial respiration, effects were abolished in *bnip3l^−/−^* mice (Figure 4I).

### 2.5 Mitochondrial Fission in *BLA* Neurons is Essential for Contextual Fear Memory Formation

**FIGURE 5:**
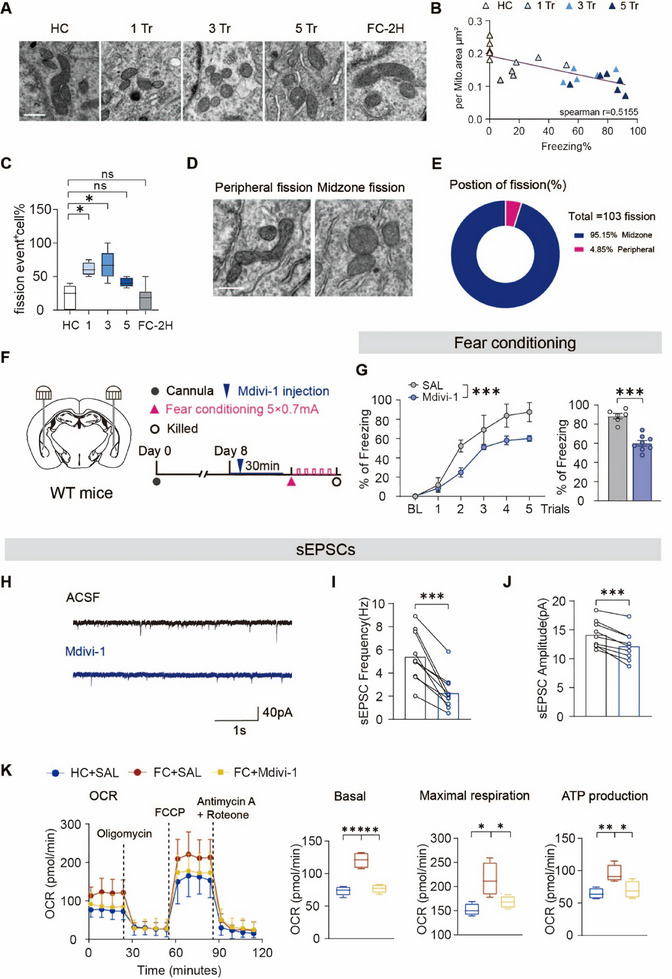
Mitochondrial fission in the BLA neurons is essential for contextual fear memory formation. (A) Representative electron microscope graphs. *n* = 6 mice per group. Scale bars = 200 nm. (B) Analyses of corrections between mitochondrial area and freezing level. *n* = 5–6 mice per group. (C) Quantitative analysis of percentage of cells undergoing mitochondrial fission. *n* = 6 mice per group. (D) Representative electron microscope graphs of mitochondrial fission. Scale bars = 100 nm. (E) Quantification of the percentage of midzone fission/peripheral fission in the BLA. *n* = 5–6 mice per group. (F) Schematic contextual fear conditioning behavior with pharmacological treatment. (G) The curve of freezing level in each trial (left) and the percentage of freezing time during the contextual fear conditioning (right). *n* = 6–8 mice per group. (H–J) Representative trace of sEPSC (H), sEPSC frequency (I), and amplitude (J) *n* = 9 cells from 3 mice. (K) Mitochondrial oxygen consumption, basal respiration, maximal respiration, ATP production, and respiratory reserve capacity. *n* = 4 mice per group. Statistical comparisons were performed as follows: one‐way repeated measures ANOVA with *post hoc* Sidak's test for B and C, two‐way repeated measures ANOVA for G (left) and K, unpaired t test for G (right) and paired t test for I and J. ^*^p < 0.05; ^**^p < 0.01; ^***^p < 0.001; n.s. vs. the indicated group.

We next asked whether mitochondrial fission in BLA neurons is essential for fear memory conditioning. TEM analysis revealed increasing neuronal mitochondrial fission events along with the trails (Tr) of conditioning in WT mice, which was recovered 2 h post fear conditioning (Figure 5A). We found a negative correlation between the freezing duration and mitochondrial size in the BLA neurons of each mouse (Figure 5B), suggesting an involvement of mitochondrial fragmentation in fear conditioning. Further analysis revealed increased mitochondrial fission events in the Tr groups, which were recovered 2 h post fear condition (Figure 5C), indicating a short‐term, physiological remodeling rather than pathological fragmentation. Approximately 95% of fissioning mitochondria underwent midzone fission (Figure 5D,E), a process enhancing ATP production without changing their calcium buffer capacity [48, 49]. Dynamin‐related protein 1 (Drp1) is a GTPase essential for mitochondria fission that is inhibited by Mdivi‐1. Mdivi‐1 administration to the bilateral BLA 30 min prior to fear conditioning reduced the phosphorylation of Drp1 at ser616 (Figure 5F; Figure S12A). Notably, Mdivi‐1 pre‐training treatment impaired contextual fear memory (Figure 5G), without affecting the locomotion (Figure S12B). Furthermore, both the frequency and amplitude of sEPSC were reduced by Mdivi‐1 (Figure 5H–J). Additionally, mice that received pre‐training injection of Mdivi‐1 had reduced mitochondrial respiration (Figure 5K), indicating that midzone fission increases healthy mitochondria to meet neuronal energy demands. These findings suggest that mitochondrial fission in the BLA neurons, driven by BNIP3L, is essential for the formation of contextual fear memory, highlighting its importance over mitophagy.

### 2.6 Light‐Driven Mitochondrial Fission in *BLA* Restores Fear Conditioning in *Bnip3l^−/−^* Mice

**FIGURE 6:**
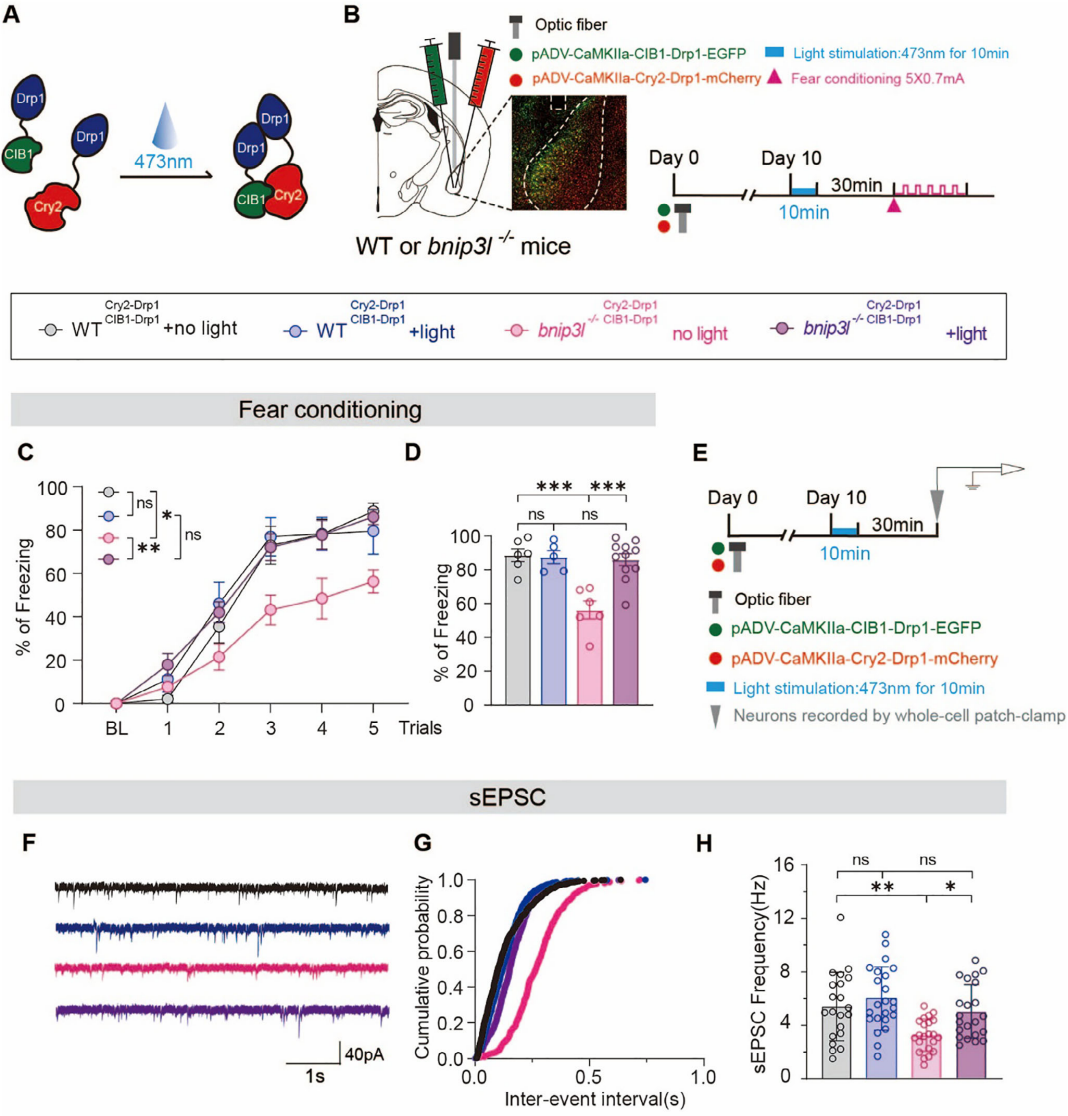
Light‐driven mitochondrial fission in the BLA restores fear conditioning deficits in *bnip3l^−/−^* mice. (A,E) Experimental schedule and timeline of light‐induced experiments. (B) Illustration of the viral injection site, viral expression, and optic fiber placement (indicated by the white dotted line) in the BLA. (C,D) The curve of freezing level in each trial (C) and the percentage of freezing time during the contextual fear conditioning (D). *n* = 5–11 mice per group. (F–H) Representative trace of sEPSC (F), cumulative plot of sEPSC interevent intervals (G), and sEPSC frequency (H). *n* = 21–25 cells from 6 mice per group. Statistical comparisons were performed as follows: two‐way repeated measures ANOVA for C‐D, G‐H. ^*^p < 0.05; ^**^p < 0.01; ^***^p < 0.001; n.s. vs. the indicated group.

To determine whether enhanced mitochondrial fission in BLA^GLU^ neurons is sufficient to restore fear memory formation in *bnip3l^−/−^* mice, a light‐inducible mitochondrial fission assay was employed. Recombinant Drp1 constructs were engineered by fusing Drp1 with either *Arabidopsis* cryptochrome 2 (Drp1‐Cry2) or cryptochrome‐interacting basic‐helix‐loop‐helix 1 (Drp1‐CIB1) [50, 51]. This optogenetic approach enables Drp1 oligomerization and subsequent mitochondrial fission upon blue light (465 nm) illumination (Figure 6A). Both WT and *bnip3l^−/−^* mice received bilateral BLA injection of AAVs encoding recombinant Drp1 under the control of *CaMKIIɑ* promoter, ensuring selective expression in excitatory neurons (Figure 6B). The effects of Drp1 oligomerization and mitochondrial fission by a 10‐min blue light illumination at BLA were confirmed (Figure S13). Blue light illumination was administered 30 min before fear conditioning. Remarkably, this intervention largely rescued the fear memory deficits observed in *bnip3l^−/−^* mice (Figure 6C,D). Additionally, whole‐cell patch‐clamp recordings in BLA^GLU^ neurons revealed that blue light‐induced mitochondrial fission restored the reduction in sEPSC frequency observed in *bnip3l^−/−^* mice (Figure 6E–H), demonstrate that BNIP3L‐driven mitochondrial fission in BLA^GLU^ neurons acts upstream of neuronal excitability and is required for fear memory formation.

### 2.7 *BNIP3L* Drives Drp1‐Dependent Mitochondrial Fission in *BLA^GLU^* Neurons

**FIGURE 7:**
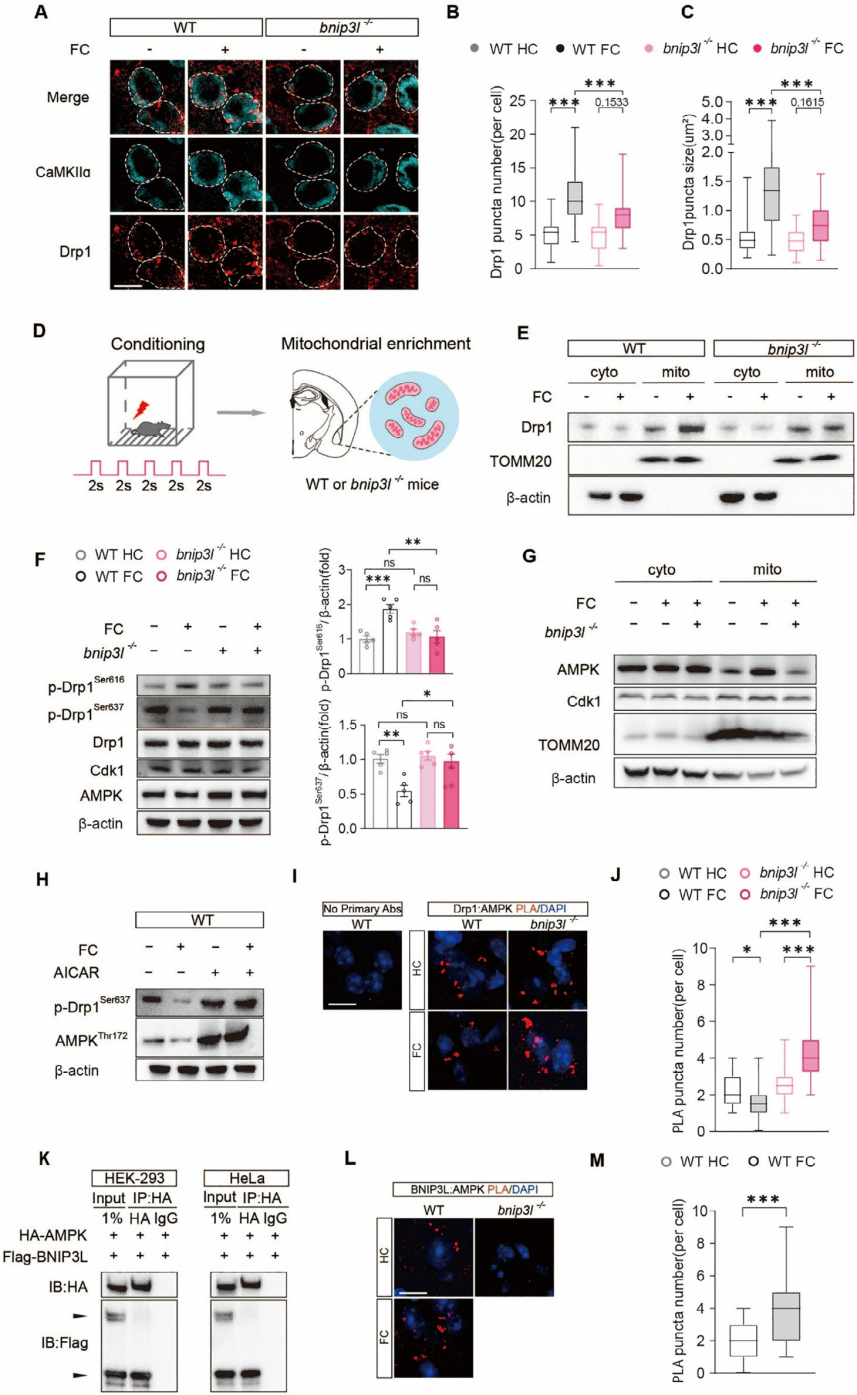
BNIP3L regulates Drp1^Ser637^ phosphorylation by competitively interacting with AMPK. (A) Representative images of mitochondria marker Hsp60 together with Drp1 in the BLA of WT or *bnip3l^−/−^* mice after fear conditioning. Scale bar = 15 µm. *n* = 3 mice per group. (B,C) Quantification for Drp1 puncta on mitochondria by puncta number (B) and size (C). (D) Schematic experimental procedure of mitochondrial fraction preparation. (E) The mitochondria fraction of WT or *bnip3l^−/−^* mice. *n* = 6 mice per group. (F) Western blot monitoring and quantitation analysis in WT or *bnip3l^−/−^* mice *n* = 6 mice per group. (G) The mitochondria fraction of WT or *bnip3l^−/−^* mice. *n* = 6 mice per group. (H) Western blot monitoring of Drp1 and AMPK of phosphorylation in WT or *bnip3l^−/−^* mice. *n* = 5–6 mice per group. (I,J) PLA detection of DRP1‐AMPK in the BLA section (I). Quantification for PLA puncta (J). *n* = 104 cells from 3 mice per group. (K) Immunoblots showing co‐immunoprecipitation of HA‐AMPK, and Flag‐BNIP3L using an anti‐HA antibody in HEK‐239 cells (left) and HeLa cells (right). (L,M) PLA detection of BNIP3L‐AMPK in the BLA section (L). Quantification for PLA puncta (M). *n* = 100 cells from 3 mice per group. Statistical comparisons were performed as follows: two‐way repeated measures ANOVA for B‐C, G. ^**^p < 0.01; ^***^p < 0.001; n.s. vs. the indicated group.

Mitochondrial fission is primarily mediated by Drp1, which is recruited to mitochondria upon activation by assembling oligomers and facilitates mitochondria division [52, 53]. We next investigated whether BNIP3L is required for Drp1‐dependent mitochondrial fission during fear conditioning. By immunostaining Drp1 in tissue slices from WT and *bnip3l^−/−^* mice, we found fear conditioning significantly increased both the number and size of Drp1 puncta on mitochondria in WT mice, whereas this effect was abolished in *bnip3l^−/−^* mice (Figure 7A–C). To further confirm these observations biochemically, we enriched mitochondrial and cytoplasmic fractions from the BLA tissues (Figure 7D). *Bnip3l* deletion significantly abolished the increasing of mitochondrial Drp1 in WT mice (Figure 7E). Collectively, these results demonstrate that BNIP3L promotes Drp1 accumulation on mitochondria in response to fear conditioning, a process that is critical for mitochondrial fission and fear memory formation.

### 2.8 *BNIP3L* Reduces the Phosphorylation of Drp1^Ser637^ by Competitively Interacting With AMPK

Phosphorylation of Drp1 at Ser616 (p‐Drp1^Ser616^) promotes, whereas phosphorylation of Drp1 at Ser637 (p‐Drp1^Ser637^) inhibits mitochondrial fission [54]. It showed that *bnip3l* deletion abolished the increase of p‐Drp1^Ser616^ and reduction of p‐Drp1^Ser637^ by FC in BLA (Figure 7F). Cdk1 and AMPK have been reported as the primary kinases for Drp1^Ser616^ and Drp1^Ser637^ phosphorylation, respectively [55], we observed a marked increase in mitochondrial localization of AMPK following fear conditioning, which was abolished in *bnip3l^−/−^* mice. In contrast, the distribution of Cdk1 remained unchanged (Figure 7G), highlighting a role of AMPK, as the primary kinase for Drp1^Ser637^, in modulating mitochondrial fission by interplaying with BNIP3L. Indeed, AICAR, an AMPK agonist, abolished FC‐reduced Drp1^ser637^ in BLA (7H). We therefore hypothesized that BNIP3L may interrupt the interaction between AMPK and Drp1. The in situ proximity ligation assay (PLA) indicated a significant increase Drp1‐AMPK interaction in the BLA from *bnip3l^−/−^* mice following fear conditioning (Figure 7I,J), supporting an interrupted interaction of AMPK with Drp1 by BNIP3L. Additionally, co‐immunoprecipitation assay revealed a direct binding of ectopic expression BNIP3L with AMPK in both HEK‐293 and HeLa cells (Figure 7K). Furthermore, PLA assay revealed increased BNIP3L‐AMPK interaction in the BLA following FC in WT mice, which was absent in *bnip3l^−/−^* mice as a negative control (Figure 7L,M). These findings indicated BNIP3L reduces the phosphorylation of Drp1^Ser637^ by competitively binding with AMPK.

## 3 Discussion

BNIP3L is a key mitophagy receptor essential for the development of multiple cell types [18, 56, 57, 58, 59]. Given its abundant expression in developing brains [15], BNIP3L deficit has been implicated in several neuropsychiatric disorders [20, 21, 37, 60]. However, its physiological role in the adult brain remains incompletely understood. In this study, we identified a previously uncharacterized function for BNIP3L in the BLA^GLU^ neurons in conditioned fear memory formation. Both global and specific ablation of *bnip3l* in BLA^GLU^ neurons resulted in significant impairments in contextual fear formation in mice. Remarkably, this function of BNIP3L was independent of its mitophagy activity. Instead, BNIP3L facilitated a rapid, Drp1‐dependent mitochondrial fission in BLA^GLU^ neurons following foot‐shock, which was essential for contextual fear memory conditioning. These findings reveal a novel and fundamental role of neuronal BNIP3L beyond its established role in mitophagy.

The BLA is a critical brain region where neutral stimuli and aversive information are integrated to encode conditioned fear memory [61, 62, 63, 64]. During fear memory conditioning, external stimuli trigger a long‐term potentiation (LTP) in the BLA glutamatergic neurons [61, 65, 66]. The excitation of these neurons activates mitochondrial fission, resulting in divided mitochondria that better accommodate presynaptic terminals or dendritic spines, thereby ensuring a localized ATP supply for synaptic activities [46, 67]. However, the molecular mechanisms driving this prompt mitochondrial fission during fear memory conditioning remain to be elucidated. We found that *bnip3l* deletion in BLA^GLU^ neurons disrupted mitochondrial fission and ATP production. This disruption impairs excitatory synaptic transmission and ultimately leads to deficits in contextual fear memory in mice. These findings uncovered the biological role for BNIP3L in regulating synaptic plasticity, at least in BLA^GLU^ neurons. BNIP3L is abundantly expressed in the adult brain (http://www.humanproteomemap.org/), including mPFC, hippocampus, and BLA, regions closely associated with conditioned fear memory [68, 69, 70, 71].

BNIP3L loss does not alter mitochondrial morphology at rest (Figure 7), likely because AMPK‐mediated inhibitory phosphorylation of Drp1 remains low and other fission‐fusion regulators compensate under baseline conditions. However, during fear conditioning, BNIP3L is specifically recruited to modulate AMPK‐Drp1 signaling, and its absence selectively disrupts activity‐dependent mitochondrial fission. We speculate that the relatively high expression of BNIP3L in BLA^GLU^ neurons is evolutionarily significant, facilitating conditioned fear memories to enable adaptive responses to stressful conditions, such as encountering predators.

Our data show that BNIP3L deletion in BLA GABAergic neurons does not affect contextual fear memory, highlighting a cell‐type‐specific role of BNIP3L. While inhibitory interneurons critically shape fear memory through network gating, their functions may rely less on mitochondrial dynamics than those of excitatory neurons. Thus, BNIP3L loss in these interneurons may not sufficiently disrupt energy regulation or synaptic function to impact behavior.

Previous studies have shown that BNIP3L‐mediated neuronal mitophagy alleviates anxiety disorder induced by chronic stress via mitigating neuroinflammation [20]. Here, we highlighted a non‐mitophagy activity of BNIP3L in regulating conditioned fear memory upon stress. Integrating these findings, BNIP3L plays multiple roles in stress adaptation. Specifically, BNIP3L‐regulated mitochondrial midzone fission may enhance neuronal excitability in adapting to acute and moderate stress, whereas BNIP3L‐mediated mitophagy may compensate for mitochondrial dysfunction caused by excessive respiration following prolonged stress‐induced neuronal activation. Our study expanded the biological significance of BNIP3L as a signaling hub in response to neuronal stress. Intriguingly, the genetic variance of BNIP3L is associated with increased vulnerability to psychiatric disorders [21], further implying the potential involvement of BNIP3L in modulating neurotransmission in critical neurons.

Our study demonstrates that Drp1 functions downstream of BNIP3L in mediating foot‐shock‐induced mitochondria fission. While Drp1 is widely recognized for its role in mitochondrial division for autophagic degradation under disease‐related stress [72, 73], its contribution to the physiological regulation of neuronal activity remains less understood. In our model, Drp1‐induced mitochondrial fission predominantly occurs in the midzone, a morphological feature indicative of energy demand rather than mitochondria degradation [49, 74, 75]. This finding aligns with our observation that Drp1‐driven midzone fission of mitochondria in BLA^GLU^ is regulated by BNIP3L through its interaction with AMPK, a key intracellular energy sensor [76, 77]. Our data support that BNIP3L competes with Drp1 for AMPK binding, thereby preventing AMPK‐mediated inhibitory phosphorylation of Drp1^Ser637^ [78, 79]. This molecular modulation by BNIP3L may facilitate a rapid response to neuronal energy demands, supporting excitation and synaptic activity. The BNIP3L related on‐demand energy supply may partly explain preserved cued fear memory in BLA^GLU^‐shBNIP3L mice, which is less metabolically demanding compared to contextual fear formation [80, 81, 82]. Collectively, we propose BNIP3L modulates Drp1‐dependent mitochondrial fission to balance autophagic mitochondria degradation and bioenergetic adaptation.

Previously, studies, including ours, have demonstrated that BNIP3L dimerization is essential for its mitophagy activity [40]. The BNIP3L dimer is required both for mitochondria fragmentation and mitophagy in epidermal keratinocytes [23]. Our data indicated that AMPK preferentially binds to the monomeric form of BNIP3L, suggesting a distinct biological role for BNIP3L monomers in regulating mitochondrial fission following neuronal activity, in addition to the established role of BNIP3L dimers in mitophagy.

In conclusion, the present study reveals a novel role of BNIP3L in regulating neuronal mitochondrial dynamics and synaptic plasticity, extending beyond its canonical function in mitophagy. We demonstrate that BNIP3L facilitates Drp1‐dependent mitochondrial fission in BLA^GLU^ neurons following foot‐shock, a process essential for contextual fear memory formation. Because BNIP3L deficiency selectively disrupts contextual fear memory while sparing general cognition, targeted enhancement of BNIP3L‐Drp1‐dependent mitochondrial dynamics may offer a way to modulate emotional‐learning circuits without broadly affecting other brain functions, shedding light on potential therapeutics for neuropsychiatric disorders associated with stress dysregulation.

## 4 Materials and Methods

### 4.1 Animals

All mice were maintained on a C57BL/6J genetic background. *Bnip3l^−/−^* mice were generously provided by Prof. Paul Ney (St. Jude Children's Research Hospital). *CaMKIIɑ‐Cre* mice (heterozygous mice Jackson Laboratory, 005359) were employed for behavioral assessments. C57BL/6J wild‐type mice (Beijing Vital River Laboratory Animal Technology Co., Ltd.) were also included in the study. The mice were housed under a 12‐h light/dark cycle (lights on at 7:00 a.m. and off at 7:00 p.m.) in a controlled environment with constant temperature and humidity, with food and water available ad libitum. All procedures were approved by the Animal Advisory Committee of Zhejiang University.

### 4.2 Behavior Assays

Mice aged 8 to 12 weeks were used for all behavioral tests. We describe them in detail in the Supporting Information.

### 4.3 Virus Information

AAV2/9‐CBG‐DIO‐EGFP‐shRNA(BNIP3L)‐WPRE(5.54 × 1012 V.G./ml), AAV2/9‐CBG‐DIO‐EGFP‐shRNA(NC)‐WPRE(5.91 × 1012 V.G./ml), pAAV‐CaMKIIɑ‐BNIP3L(delet35‐38aa)‐3×FLAGP2A‐EGFP(8.0 × 1012 V.G./ml), pAAV‐CaMKIIɑ‐BNIP3L3×FLAG‐P2A‐EGFP(2.74 × 1012 V.G./ml), pADV‐CaMKIIɑ‐CIB1Drp1‐EGFP(6.32 × 1010 V.G./ml), and pADV‐CaMKIIɑ‐Cry2‐Drp1‐mCherry(8.69 × 1010 V.G./ml) were purchased from OBio BiotechCo., Ltd (Shanghai, China). AAV2/9‐CMV‐DIO‐EGFP‐shRNA(ATG7)‐WPRE‐hGH‐polyA(5.0 × 1012 V.G./ml) and AAV2/9‐CMV‐DIO‐EGFP‐shRNA(scramble)‐WPRE‐hGH‐polyA (5.1 × 1013 V.G./ml) were purchased from BrainVTA(Wuhan, China). All viruses were aliquoted and stored at −80°C until use.

### 4.4 Stereotaxic Surgery, Cannula and Optical Fiber Implantation

Stereotaxic surgery and fiber implantation were performed according to previously described protocols [35, 76] and are resumed in the Supporting Information.

#### 4.4.1 Immunostaining

Mice were perfused with PBS followed by 4% paraformaldehyde; brains were post‐fixed 4 h, dehydrated in 30% sucrose, cryosectioned at 20 µm, and stored at −80°C. Sections were washed with PBS, antigen‐retrieved in 10 mm citrate buffer (95–100°C, 10 min), permeabilized with 0.1% Triton X‐100, blocked with 5% donkey serum, incubated overnight at 4°C with primary antibodies, followed by AlexaFluor‐conjugated secondary antibodies (1:400, Jackson ImmunoResearch) for 2 h, mounted with DAPI‐containing Fluoroshield, and imaged on confocal microscopes (Leica SP8 or Zeiss LSM880).

Primary antibodies (1:400): anti‐c‐Fos (ab209794, Abcam), anti‐CaMKIIɑ (ab134041, Abcam), anti‐GABA (A0310, Sigma), anti‐GFP (AB13970, Abcam), anti‐RFP (PM005, MBL), anti‐Hsp60 (12165, CST), anti‐Drp1 (8570S, CST).

Secondary antibodies (1:400): anti‐rabbit Alexa 594 (A32740, Thermo), anti‐rabbit Alexa 647 (A32733, Thermo), goat anti‐chicken Alexa 488 (A11039, Thermo).

More details are described in the Supporting Information.

#### 4.4.2 Electrophysiology

Electrophysiological recordings were conducted in whole‐cell mode for the detection of sEPSCs. Whole‐cell patch‐clamp recordings were performed to detect sEPSCs using pipettes (5‐8 MΩ) filled with K^+^‐based internal solution (135 K‐gluconate, 4 KCl, 10 HEPES, 1 EGTA, 4 Mg‐ATP, 0.4 Na‐GTP, 10 Tris‐2‐Phosphocreatine, pH 7.22). sEPSCs were recorded at −70 mV, and action potential thresholds were measured under current clamp with 20 pA incremental currents. Data were analyzed using Clampfit 11.2 and Mini Analysis. More details are described in the Supporting Information.

### 4.5 Mitochondrial Respiration Measurements

BLA tissue punches (500 µm, Rapid‐core) were transferred into a biopsy chamber with oxygenated ACSF and loaded into an XFe96 microplate (180 µL/well). Plates were incubated at 37°C for 60 min. Seahorse XFe96 sensor cartridges were hydrated, loaded with drugs, and calibrated before assay. Drugs were sequentially delivered to achieve final concentrations: Oligomycin 25 µm; FCCP + pyruvate 7.5 µm + 7.5 mm; Antimycin A + rotenone 5 µm each. Sampling times allowed steady‐state effects. Wells with low basal OCR (<20 pmol/min) or no response to FCCP/pyruvate were excluded. More details are described in the Supporting Information.

#### 4.5.1 Proximity Ligation Assay (PLA)

In situ PLA was performed on 20 µm BLA brain sections using the Duolink kit (DUO92101, Sigma). Mice were perfused with 4% paraformaldehyde, brains cryoprotected in 30% sucrose, and sections mounted on slides. Sections were blocked (Duolink, 37°C, 30 min), incubated overnight at 4°C with anti‐AMPKɑ1/ɑ2 (A27099, Abclonal, 1:100) and either anti‐Drp1 (8570, CST, 1:100) or anti‐BNIP3L (12396, CST, 1:100), followed by anti‐rat MINUS and anti‐goat PLUS probes (37°C, 30 min), ligation and amplification with Duolink far‐red reagent, and mounted with DAPI. See more details in the Supporting Information.

### 4.6 Western Blot Analysis and Immunoprecipitation

Cell and brain samples were homogenized in RIPA buffer, centrifuged at 12,000 × g for 10 min, and 40 µg protein was separated on 4–12% SDS‐PAGE gels and transferred to nitrocellulose membranes. Membranes were blocked with 5% milk and incubated overnight at 4°C with primary antibodies: anti‐SQSTM1/p62 (A19700, Abclonal), anti‐LC3B (L7543, Sigma), anti‐TOMM20 (A6774, Abclonal), anti‐β‐actin (AC006, Abclonal), anti‐ATG7 (8558T, CST), anti‐Drp1 (8570, CST), anti‐Drp1 Ser616 (4494, CST), anti‐Drp1 Ser637 (4867, CST), anti‐Cdk1 (A11420, Abclonal), anti‐AMPK (A27740, Abclonal), anti‐AMPK Thr172 (AP1441, Abclonal), anti‐BNIP3L/NIX (12396, CST), anti‐DYKDDDDK‐Tag (14793, CST), and anti‐HA Tag (3724, CST), followed by HRP‐conjugated secondary antibodies (1:3000, Abclonal). Signals were detected with ECL (PK10001, Proteintech) and quantified using Image Pro Plus 6.0. For immunoprecipitation, HEK‐293T and HeLa cells were lysed in RIPA buffer with protease inhibitors, incubated with anti‐HA antibody (3724, CST) and Protein A/G magnetic beads (HY‐K0202, MCE) overnight at 4°C, and eluted with 1× loading buffer for SDS‐PAGE. See more details in the Supporting Information.

### 4.7 Transmission Electron Microscope

TEM was conducted by the center of Electron Microscopy of Zhejiang University. See details in the Supporting Information.

### 4.8 Quantification and Statistical Analysis

All data are expressed as mean ± SEM. The number of experimental replicates (n) is specified in the figure legends and corresponds to the number of independently treated experimental subjects in each condition. To assess data distribution, the Kolmogorov‐Smirnov normality test was performed. For comparisons between two groups, an unpaired t‐test or a paired two‐tailed t‐test was applied, while one‐way ANOVA was used for analyzing differences among three or more groups under the assumption of normal distribution. Depending on the experimental design, one‐way repeated measures ANOVA, two‐way ANOVA, and two‐way repeated measures ANOVA were appropriately utilized. Statistical analyses were conducted using Prism (version 9.0), with a significance threshold set at 0.05. Statistical significance is indicated in all figures as *p < 0.05, **p < 0.01, and ***p < 0.001.

## Funding

Key Research and Development Program of Zhejiang Province (2025C02109), National Natural Science Foundation of China (82373923, 82173792), and the Jinhua Science and Technology Plan Projects (2023‐3‐170).

## Conflicts of Interest

The authors declare no conflict of interest.

## Supporting information




**Supporting File**: advs73983‐sup‐0001‐SuppMat.docx

## Data Availability

The data that support the findings of this study are available on request from the corresponding author. The data are not publicly available due to privacy or ethical restrictions.
