# Supplementary material for: A Non‐Mitophagy Activity of BNIP3L/NIX in Amygdala Glutamatergic Neurons is Essential for Contextual Fear Memory Formation
Source: Adv Sci (Weinh). 2026 Jan 25;13(18):e17585. doi: 10.1002/advs.202517585 (PMC13042501; doi:10.1002/advs.202517585)
Supplement: Supplementary file 1 — Supporting File: advs73983‐sup‐0001‐SuppMat.docx [file ADVS-13-e17585-s001.docx]

Supporting Information

**A Non-Mitophagy Activity of BNIP3L/NIX in Amygdala Glutaminergic Neurons is Essential for Contextual Fear Memory Formation**

*Xingxian Zhang^1^, Xinlei Mo^1^, Xinyu Zhou^1^, Xiaoliang Liu^1^, Guizhi Li^2^, Songhui Hu^4^, Yangyang Lu^1^, Chenze Zhu^1^, Jinxi Feng^1^, Zhitong Chen^1^, Weiwei Hu^3^, Yihui Cui^5^, Zhong Chen^1,6,^*, Xiangnan Zhang^1,2,4^**

**Supplementary Methods and Materials**

***Behavioral Assays***

***Contextual Fear Conditioning Test***

The fear conditioning test was conducted in a square-shaped chamber equipped with a metal grid floor for shock delivery (Coulbourn Instruments, USA). To habituate the mice, they were placed in the apparatus without exposure to shocks for 5 minutes on the day prior to training. A two-day protocol was implemented to assess contextual fear memory.

On Day 1 (Conditioning), mice were introduced to the foot-shock box and allowed to explore freely for 2 minutes. Subsequently, they underwent five tone-shock pairings. (0.7 mA, 2 s). Following the final foot-shock, mice remained in the chamber for additional two minutes. On day 2 (24 hours later, contextual memory recall), the mice were reintroduced to the same foot-shock box and allowed to explore for 5 minutes without exposure to shocks. The box was cleaned with 70% ethanol between subjects.

All behavioral sessions were recorded on video, and freezing behavior (immobility lasting ≥1 s) was automatically analyzed using Freezeframe software and manually verified in a blinded manner.

***Body Temperature Measurement***
Mouse body temperature was measured using a digital rectal thermometer designed for rodents. The mice were gently restrained, and the probe was gently inserted to a consistent depth into the anus. The probe was disinfected with 70% ethanol between trials.

***Hargreaves Test***
Mice were placed in a transparent Plexiglas chamber with a glass bottom and allowed to acclimate for 1 hour before testing. A radiant heat source (Ugo Basile, 20 W) was positioned beneath the glass, targeting the plantar surface of the hind paw. Paw withdrawal latency was automatically recorded, with minimum and maximum cutoff times set at 1 and 20 seconds, respectively. Each paw was tested in two separate trials, with a 15-minute interval between them. The average of both trials was used for analysis.

***Von-Frey Test***Mice were placed in an elevated cage with a wire mesh floor for 30 minutes to acclimate before testing. On Days 0, 7, 14, 21, and 28, a series of von Frey filaments (numbers 2 through 9) with bending forces ranging from 0.02 to 1.4 g were applied to the plantar surface of the hind paw for 3 seconds. The withdrawal threshold was determined using the "up and down" method, which involves sequentially increasing and decreasing the stimulus strength. Each hind paw was tested five times, with a 5-minute interval between trials. In the fifth test, if the mouse exhibited a withdrawal response, the filament number was reduced by 0.5; if no response occurred, 0.5 was added. The mechanical pain threshold was calculated using the following formula:

PWT_force_=10^(x×F+B)^

where F is the final von Frey filament number, x = 0.240, and B = -2.00. The average withdrawal threshold from ten trials per mouse was used for analysis.

***Repetitive Behaviors***Mice were placed in a clean cage with fresh bedding for a 30-minute acclimation period. The incidence of repetitive behaviors, including digging, grooming, and rearing, was recorded on video and measured over a 10-minute observation period. Digging behavior was defined as the coordinated use of the forelegs or hind legs to displace or excavate bedding materials. Grooming behavior included actions such as stroking or scratching the face, head, or body with the forelimbs, as well as licking various body parts. Rearing was characterized by the mouse standing on its hind legs, either at the corner of the cage or along the walls.

***Prepulse Inhibition***

The PPI test was conducted using SR-LAB startle chambers. Mice were first acclimated in the chambers for 5 minutes, followed by five 20-ms startle pulses at 120 dB. Next, six series of tests were performed to measure PPI, each consisting of eight different stimulus conditions presented in a pseudorandom order: no stimulus, startle tone only, pulse tone only (74, 78, or 82 dB), and pulse paired with a startle tone. Following these trials, mice were exposed to five additional 20-ms, 120 dB startle pulses. The maximum amplitude of the startle response for each trial was recorded, and the average of the six tests was used for data analysis. PPI was calculated using the following formula:

PPI% = [1- (average response to prepulse before startle stimulus/average response to startle stimulus)] × 100%.

***Rotarod***

The mice were tested using a rotarod fatigue apparatus. Over a 5-minute period, the rotational speed was gradually increased from 5 rpm/min to 40 rpm/min, and the latency to fall was recorded. If the mouse did not fall within 5 minutes, a maximum fall time of 300 seconds was assigned. Each mouse underwent 8 trials, with a 20-minute interval between each trial.

***Open Field Test***The open field test was conducted in a 45 cm × 45 cm × 45 cm arena, with a central zone demarcated by lines 11 cm from the walls. Mice were placed at the center of the arena and allowed to explore for either 30 minutes. Their movements were tracked and analyzed using the ANY-maze video tracking system.

***Light-Dark Box***

The light-dark box was partitioned into a dark compartment (∼5 lux, 30 × 20 × 25 cm) and a light compartment (∼600 lux, 15 × 20 × 25 cm), connected by a small opening. Mice were placed at the center of the light compartment and allowed to move freely between the two compartments for 5 minutes. During this time, their movements were automatically tracked and analyzed using the ANY-maze system. The number of entries into the light compartment and the total time spent in the light compartment were recorded.

***Sucrose Preference Test***

Mice were individually housed and habituated to two water bottles for 2 days (W/W), followed by two bottles of 1% sucrose solution for 2 days (S/S). Subsequently, the mice were given a free choice between water and 1% sucrose for 2 days (W/S). To minimize position bias, the positions of the bottles were swapped daily. The daily consumption of water and sucrose solution from each bottle was measured by weight. The sucrose preference percentage was calculated using the following formula:
Sucrose preference%= (Total sucrose consumption / (Total water consumption +Total sucrose consumption) × 100%.

***Tail Suspension Test***Mice were suspended 60 cm above the ground by their tails using tape at the tail tip for 6 minutes. Animal behaviors were recorded from the side. The immobile time, defined as the absence of any movement, was manually assessed during the last 4 minutes of the test.

***Forced Swimming Test***Mice were placed in a transparent cylinder (20 cm in diameter, 30 cm in height) filled with water (23°C-25°C) for 6 minutes. Animal behaviors were filmed from the side. The immobile time, defined as the period when the animals remained floating or motionless, with only minimal movements necessary to maintain balance, was manually recorded during the final 4 minutes of the test.

***Y-maze Spontaneous Alternation Test***

The test was conducted in a Y-shaped maze consisting of three white, opaque plastic arms (labeled A, B, and C), arranged at 120° angles. Mice were placed at the center of the maze and allowed to explore freely for 8 minutes. Their movements were recorded via video. The total number of arm entries (Total) and the number of triads (Triads) were manually counted in a blind manner. An entry was defined as the animal's body center crossing into an arm. Spontaneous alternation ratio = (Triads/total-2) × 100%.

***Novel Object Recognition Test***

Mice were acclimated to the open field apparatus for 10 minutes the day prior to testing. On the following day, two identical objects were placed in the apparatus, and the mice were allowed to freely explore for 10 minutes. Thirty minutes later, one of the objects was replaced with a novel one, and the mice were given another 5 minutes to explore. During both sessions, the mice's movements were recorded by video, and the time spent exploring the familiar (F) and novel (N) objects was manually measured in a blind manner. Exploration was defined as when the mouse’s nose was within 1 cm of the object, excluding time spent climbing on the object. The discrimination index= [(N-F)/(N+F)] × 100%.

***Morris Water Maze Test***

The Morris water maze test was conducted in a circular pool filled with water (22°C-24°C) and rendered opaque with non-toxic tempera paint. The pool was divided into four quadrants (NW, SW, NE, and SE), each marked with distinct visual cues on the pool walls. A round platform (10 cm in diameter) was submerged 1.0 cm below the water's surface in the center of the SE quadrant. Animal movements were tracked via a video monitoring system (Coulbourn Instruments, USA) positioned above the pool throughout the trials.

The test consisted of five days of training followed by a single test day. On days 1-5, mice underwent four trials per day with 20-minute inter-trial intervals, during which they were trained to locate the hidden platform. For each trial, the mice were gently introduced into the water facing the pool wall from different quadrants and allowed to swim freely for up to 60 seconds. If a mouse located the platform and remained on it for 10 seconds within 60 seconds, the trial ended automatically. The latency to reach the platform was recorded. If the mouse failed to find the platform within the time limit, it was gently guided to the platform, where it remained for 10 seconds, and the latency was recorded as 60 seconds.

On day 6, the platform was removed, and the mice were introduced to the water from the NW quadrant. They were allowed to swim freely for 60 seconds, and the following parameters were recorded: the number of crossings over the platform area, the percentage of time spent in the target quadrant, and the average swimming speed.

***Stereotaxic Surgery, Cannula and Optical Fiber Implantation***

8-weeks-old mice were anesthetized with isoflurane and positioned in a stereotaxic apparatus (RWD Life Science, China). A 1 mL syringe (Gaoge Industrial and Trading Co., Ltd, Shanghai, China), controlled by a micropump (Micro 4, World Precision Instruments, USA), delivered viral solutions at a rate of 60 nL/min. A total volume of 150 nL was injected into the BLA and 200 nL into the vCA1). To minimize reflux, the needle remained in place for 5 minutes post-injection before being gradually withdrawn. Bilateral injections were performed at the following stereotaxic coordinates (relative to bregma): BLA—AP: -1.1 mm, ML: ±3.5 mm, DV: -4.8 mm; vCA1—AP: -3.2 mm, ML: ±3.3 mm, DV: -4.2 mm. Mice were housed for three weeks post-injection to allow for viral expression before behavioral assessments. Following behavioral tests, brain sections were examined under a fluorescence microscope to confirm GFP expression, and data from mice with inaccurate injection sites were excluded.

For intra-BLA drug administration and blue-light stimulation, cannulas or optical fibers (RWD Life Science, China) were implanted at the following coordinates (relative to bregma): AP: -1.1 mm, ML: ±3.5 mm, DV: -4.6 mm. Cannulas were inserted gradually and secured to the skull using four anchor screws and a layer of dental cement. Mice were allowed to recover in their home cages for 1–2 weeks before experimental procedures. At the conclusion of the study, cannula placements were verified, and data from mice with misaligned implantations were excluded from analysis.

***Immunostaining***
Mice were sacrificed and perfused with 0.01 M PBS followed by 4% paraformaldehyde in PBS (pH 7.4). The brains were quickly dissected and further fixed in 4% paraformaldehyde for an additional 4 hours, after which they were dehydrated through 30% sucrose solutions. Cryogenic brain sections were made at a thickness of 20 μm, which were stored at -80°C until use.

Brain slices were washed three times with PBS for 5 minutes each. To prepare the antigen retrieval buffer, 41 mL of solution A (0.1 M sodium citrate: 29.41 g sodium citrate dihydrate in 1000 mL ddH_2_O) and 9 mL of solution B (0.1 M citric acid: 21.01 g citric acid monohydrate in 1000 mL ddH_2_O) were combined in 450 mL ddH_2_O to make a 10 mM antigen retrieval buffer. The slices were immersed in the preheated antigen retrieval buffer and incubated at 95-100°C for 10 minutes. After cooling to room temperature, the slices were permeabilized with 0.1% Triton X-100 in PBS for 15 minutes.

Blocking was performed by incubating the slices with normal donkey serum (5%) in PBS for 1 hour at room temperature. The slices were then incubated overnight at 4°C with primary antibodies. Afterward, the slices were incubated with AlexaFluor-conjugated secondary antibodies (1:400, Jackson ImmunoResearch Laboratories, USA) for 2 hours at room temperature. After mounting with FluoroshieldTM containing DAPI (Sigma-Aldrich, USA), the slices were observed using a confocal microscope (Leica SP8, Germany, or Zeiss LSM880 with Airyscan, Germany). Image analysis was performed using ImageJ in a blinded manner.

The primary antibodies used were as follows: anti-c-Fos (ab209794, abcam, 1:400), anti-CaMKIIɑ (ab134041, Abcam, 1:400), anti-GABA (A0310, Sigma, 1:400), anti-GFP (AB13970, Abcam, 1:400), anti-RFP (PM005, MBL, 1:400), anti-Hsp60 (12165, Cell Signaling Technology, 1:400), and anti-Drp1 (8570S, Cell Signaling Technology, 1:400). The secondary antibodies conjugated with anti-rabbit Alexa 594 (A32740, Thermo Fisher Scientific, 1:400), anti-rabbit Alexa 647 (A32733, Thermo Fisher Scientific, 1:400), Goat anti-chicken Alexa 488 (A11039, Thermo Fisher Scientific, 1:400).

***Slice Preparation***

The mice were rapidly perfused with ice-cold artificial cerebrospinal fluid (ACSF) containing (in mmol/L): 194 sucrose, 30 NaCl, 26 NaHCO_3_, 4.5 KCl, 1.2 NaH_2_PO_4_, 0.2 CaCl_2_, 4 MgCl_2_, and 10 glucose, oxygenated with 95% O_2_ and 5% CO_2_. The brain was then promptly transferred to a vibratome (VT1000S, Leica) to obtain 300 μm coronal slices containing the BLA region. For electrophysiological recordings, the slices were placed in a chamber containing ACSF (in mmol/L) :119 NaCl, 26 NaHCO_3_, 2.5 KCl, 1 NaH_2_PO_4_, 2.5 CaCl_2_, 1.3 MgCl_2_, and 11 glucose. And oxygenated with 95% O_2_ and 5% CO_2_. The slices were incubated at 34°C for 30 minutes, followed by room temperature incubation for 1 hour before recording.

***Electrophysiology***

Electrophysiological recordings were conducted in whole-cell mode for the detection of sEPSCs. For recordings of spontaneous synaptic currents, patch pipettes (5–8 MΩ) were filled with potassium-based internal solution containing (in mmol/L): 135 K-gluconate, 4 KCl, 10 HEPES, 1 EGTA, 4 Mg-ATP, 0.4 Na-GTP, and 10 Tris-2-Phosphocreatine (pH 7.22). To record the action potential threshold, episodic currents were injected under the current clamp configuration in 20pA increments from 0 pA to depolarizing 280 pA. sEPSCs were recorded at a holding potential of -70 mV. Data analysis was performed using Clampfit 11.2 and Mini Analysis.

***Mitochondrial Respiration Measurements***

Brain tissue sections were individually transferred into a biopsy chamber containing freshly oxygenated ACSF. BLA tissue were collected using a stainless-steel punch needle (Rapid-core, 500 μm diameter). The tissue samples were directly ejected into an XFe96 Cell Culture Microplate (103708-100; Agilent Technologies) according to a pre-established layout. Each well contained 180 µL of assay media (room temperature ACSF). After loading the tissue samples, each well was visually inspected to ensure that the tissue sections were submerged and properly centered at the bottom. The microplate was incubated at 37°C for approximately 60 minutes. During this incubation, drugs for the assay were prepared in ACSF and loaded into the respective injection ports of a hydrated Seahorse XFe96 Extracellular Flux Assay sensor cartridge (overnight hydration in distilled water, followed by exchange with XF Calibrant solution 1 hours before assay). The sensor cartridge was then calibrated in the Seahorse analyzer, after which the calibration plate was replaced by the plate containing the tissue punches, and the assay protocol was initiated.

The assay drugs were sequentially delivered to achieve the following final concentrations: Oligomycin (25 μmol/L); FCCP + pyruvate (7.5 μmol/L + 7.5 mmol/L); Antimycin A + rotenone (5 μmol/L + 5 μmol/L). These concentrations were optimized based on prior experiments to achieve the desired pharmacological effects. The sampling duration for each condition was selected to allow the drug effects to reach a steady state. Wells showing low basal activity (< 20 pmol/min OCR) or failing to respond to FCCP/pyruvate treatment were excluded from analysis.

***Proximity Ligation Assay (PLA)***

In situ PLA was conducted on mouse brain sections using the Duolink in situ detection kit (DUO92101, Sigma-Aldrich) following the manufacturer's instructions. Mice were anesthetized with chloral hydrate (400 mg/kg, intraperitoneally) and perfused with 4% paraformaldehyde. The brains were then removed, cryoprotected with 30% sucrose, and cut into 20 µm thick coronal sections containing the BLA region, which were subsequently mounted on microscope slides. The brain sections were blocked with Duolink blocking solution for 30 minutes at 37°C, followed by overnight incubation at 4°C with mouse monoclonal anti-AMPKɑ1/ɑ2 (A27099, abclonal, 1:100) and rabbit monoclonal anti-Drp1 (8570, Signaling Technology, 1:100) or rabbit monoclonal anti-BNIP3L (12396, Signaling Technology, 1:100) antibodies. After washing, samples were incubated with anti-rat MINUS and anti-goat PLUS probes for 30 minutes at 37°C. The ligation and amplification reactions were then performed using the Duolink far-red detection reagent. Brain sections were mounted with DAPI.

***Transfection***

The plasmids were transfected into HEK-293T and HeLa cells according to the jet PRIME (Polyplus, nl14-15) protocol.

***Western Blot Analysis***

Cell and brain tissue samples were homogenized in RIPA buffer. The supernatants were collected following centrifugation at 12,000×g for 10 minutes. A 40-μg aliquot of protein from each sample was separated using 4-12% SDS-PAGE gels and transferred to a nitrocellulose membrane. The membrane was then blocked with 5% nonfat milk in PBS (pH 7.4) and incubated overnight at 4°C with primary antibodies. Afterward, the membrane was incubated with secondary antibodies at room temperature for 2 hours.

The following primary antibodies were used: anti-SQSTM1/p62 (A19700, abclonal, 1:1000), anti-LC3B antibody (L7543, Sigma, 1:1000), anti-TOMM20 (A6774, abclonal, 1:1000), anti-β-actin antibody (AC006, abclonal, 1:10000), anti-ATG7 antibody (8558T, Signaling Technology, 1:1000), anti-Drp1 (8570, Signaling Technology, 1:1000), anti-Drp1 Ser616 (4494, Signaling Technology, 1:1000), anti-Drp1 Ser637 (4867, Signaling Technology, 1:1000), anti-Cdk1 (A11420, abclonal, 1:1000), anti-AMPK (A27740, abclonal, 1:1000), anti-AMPK Thr172 (AP1441, abclonal, 1:1000), anti-BNIP3L/NIX (12396, Cell Signaling Technology, 1:1000), anti-DYKDDDDK-Tag (14793, Cell Signaling Technology, 1:1000), anti-HA Tag (3724, Signaling Technology, 1:1000). And secondary antibodies conjugated with HRP (1:3000; ABclonal) for either rabbit or mouse IgG. Digital images were captured using a gel documentation system (Tanon 5200, China) following exposure to ECL reagent (PK10001, Proteintech). Protein expression was quantified using Image Pro Plus (version 6.0) in a blind manner.

***Immunoprecipitation***

For immunoprecipitation, HEK-293T and HeLa cells were transiently transfected. After 24 hours, cells were lysed in RIPA buffer supplemented with a protease inhibitor cocktail. Immunoprecipitation was carried out using Protein A/G Magnetic Beads (HY-K0202, MCE), For HA immunoprecipitation, the supernatant was incubated with anti-HA antibody (3724, Cell Signaling Technology, 1:100) overnight at 4°C under gentle rotation, and the bound proteins were eluted with 60 μL of 1 × loading buffer (20315ES20, Yeasen).

***Transmission Electron Microscope***

Mice were anesthetized and perfused, and the brain was quickly removed and immersed in a pre-cooled fixative solution containing 2.5% glutaraldehyde overnight at 4°C. Following a rinse in 0.1 M PBS, the tissues were post-fixed in 2.5% glutaraldehyde for 2 hours, washed twice in 0.1 M PBS, and then fixed with 1% osmium tetroxide for 5 minutes at room temperature.

Subsequently, the samples were dehydrated through a graded series of ethanol solutions (50%, 70%, and 90%) for 10 minutes each, followed by a 10-minute treatment with a 90% ethanol and 90% acetone mixture, and a final dehydration step in 100% acetone for 10 minutes. The samples were then stained with entrapment media EPON812 in 100% acetone for 2 hours, infiltrated, and embedded in EPON812 before polymerizing at 60°C for 24 hours. Thin sections (200 nm) of the BLA region were cut using a diamond knife, post-stained with uranyl acetate and lead citrate, and examined under a Tecnai G2 F20 transmission electron microscope (FEI) operating at 80 kV.

**
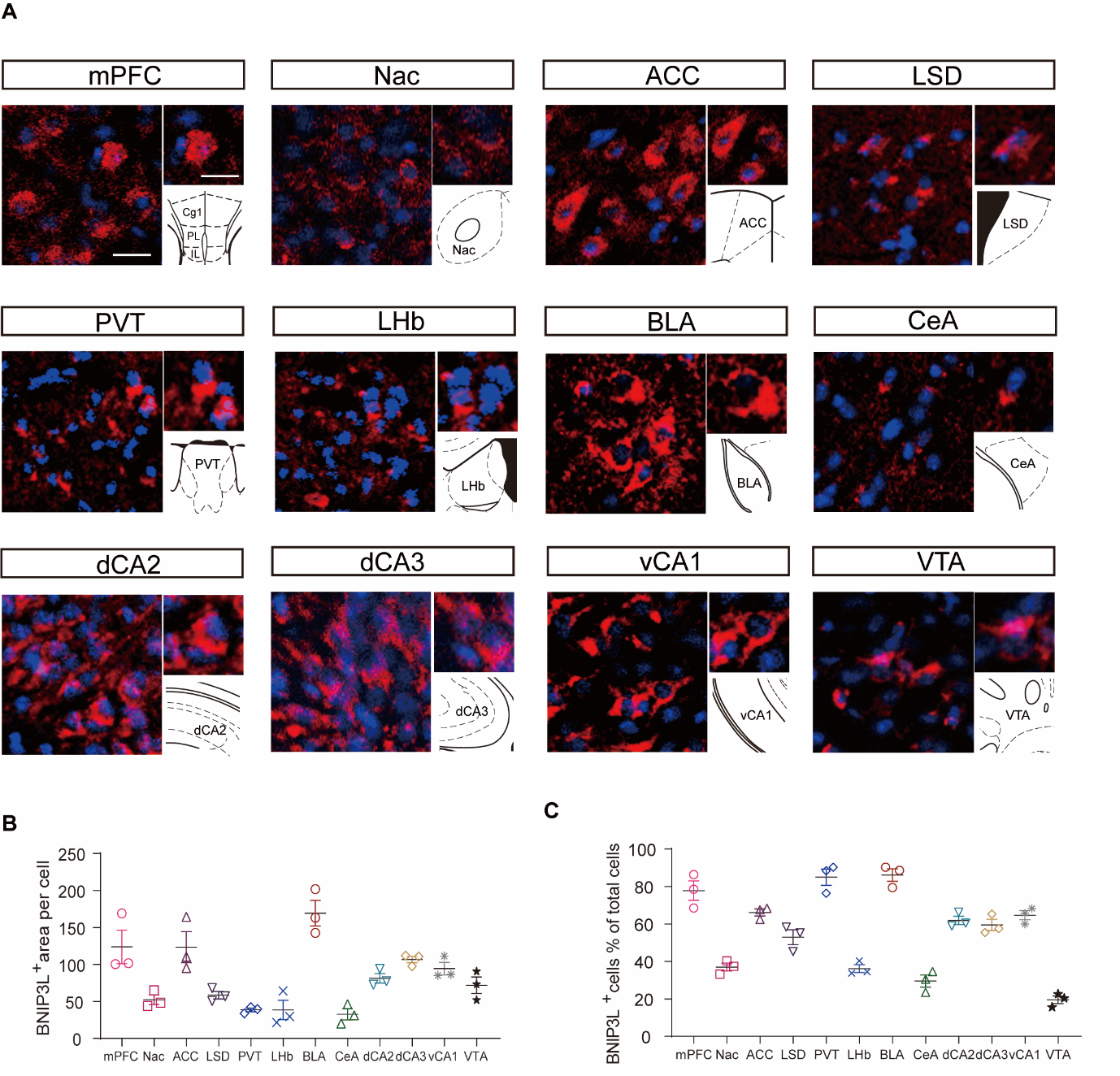
Supplemental figure**

**Figure S1.** The expression patterns of BNIP3L in adult mice brain. (**A**) Representative images of BNIP3L expressing in WT mice. Left: Scale bar = 30 μm. Right: scale bar = 15 μm. *n* = 3 mice. (**B**) Quantitative analysis of BNIP3L protein expression in BNIP3L^+^ cell of the indicated areas. (**C**) The percentage of BNIP3L^+^ cell in total cells of the indicated areas. The abbreviations used are as follows: mPFC, medial prefrontal cortex; Nac, nucleus accumbens; ACC, anterior cingulate cortex; LSD, lateral septum; BLA, basolateral amygdala, CeA, central amygdala; dCA2, dorsal CA2 region of the hippocampus; dCA3, dorsal CA3 region of the hippocampus; vCA1, ventral CA1 region of the hippocampus; LHb, lateral habenula; PVT, paraventricular nucleus of the thalamus; VTA, ventral tegmental area. Data are expressed as
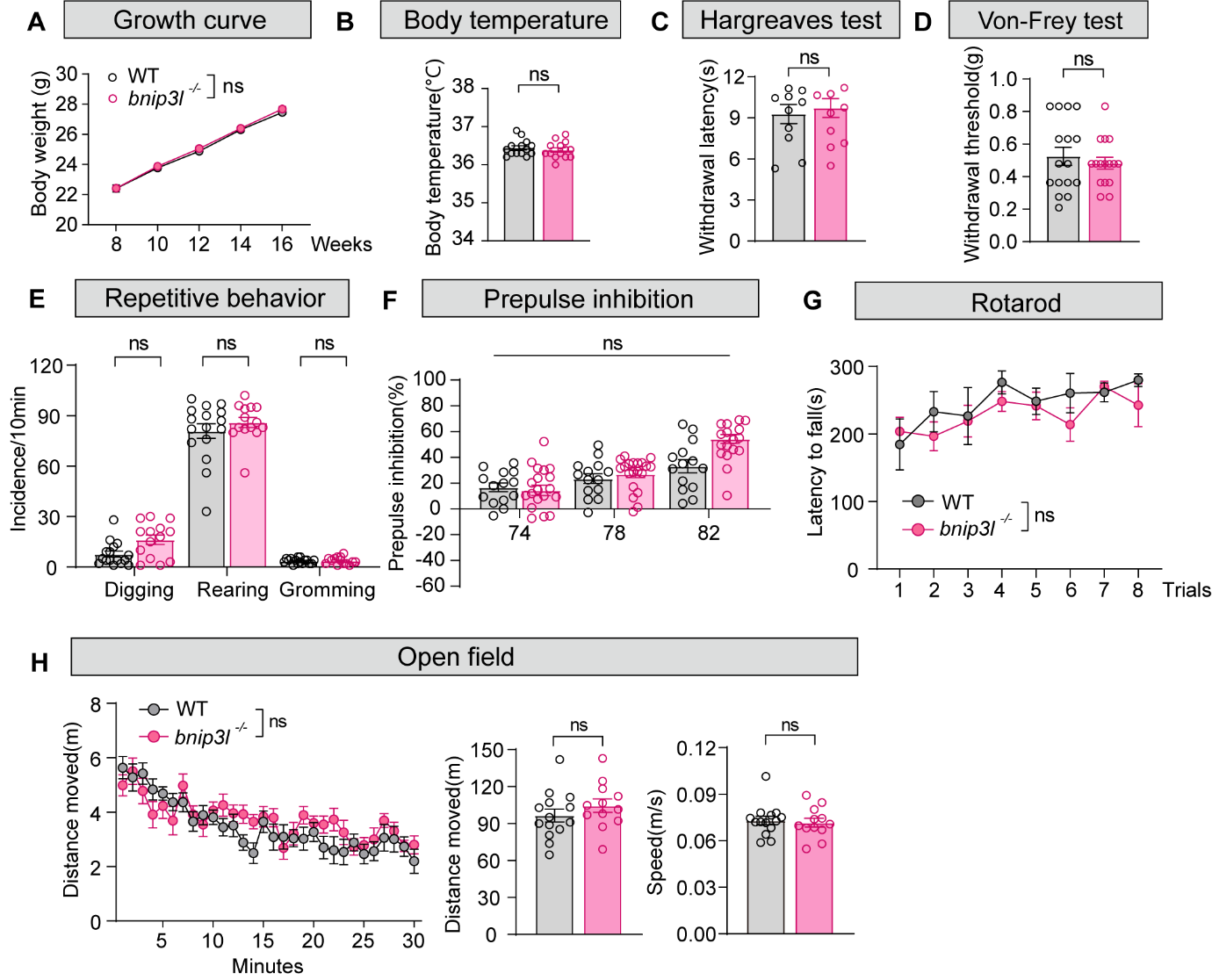
mean ± SEM.

**Figure S2.** The body weight, body temperature, pain sensitivity, repetitive behavior, sensorimotor gating and locomotor activity were not altered in *bnip3l^-/-^* mice. (**A**) Body weight from 8 to 16 weeks of age. Two-way repeated measures ANOVA, ns nonsignificant. (**B**) Body temperature tested by a rectal thermometer. Unpaired two tailed t test, ns nonsignificant. (**C**) Latency for paw withdrawal in the Hargreaves test. Unpaired two tailed t test, ns nonsignificant. (**D**) Mechanical allodynia paw withdrawal in the Von-Frey test. Unpaired two tailed t test, ns nonsignificant. (**E**) Incidence of digging, grooming, and rearing across 10 min. Unpaired two tailed t test, ns nonsignificant. (**F**) Percentage of prepulse inhibition of the auditory startle reflex under 74, 78, and 82 dB prepulse intensity. Two-way repeated measures ANOVA, ns nonsignificant. (**G**) Latency to fall in the rotarod test over 8 trials. Two-way repeated measures ANOVA, ns nonsignificant. (**H**) Locomotor activity depicted in 5-min segments (left), total distance moved (middle) and speed (right) in the open field over a 30-min period. Locomotor activity depicted in 5-min segments: two-way repeated measures ANOVA, ns nonsignificant. Total distance moved and speed: unpaired two tailed t test, ns nonsignificant. *n* = 10-12 mice per group. Data are expressed as mean ± SEM.


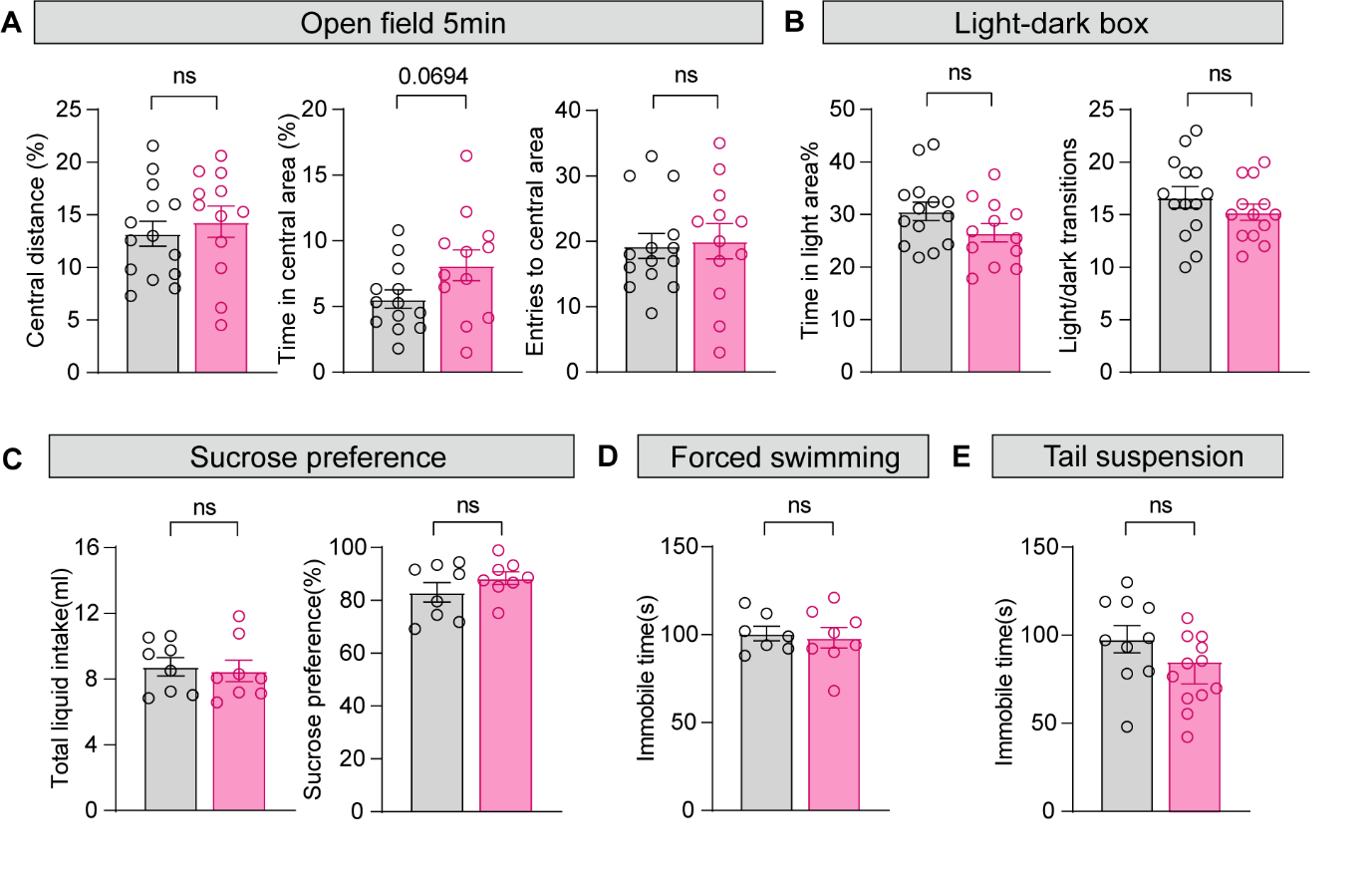
**Figure S3.** The anxiety- and depression-like behavior were not altered in *bnip3l^-/-^* mice. (**A**) The percentage of distance moved and the percentage of time spent in central zone and the entries to central area in the first 5 min during a 30 min-open field test. Unpaired two tailed t test, ns nonsignificant. (**B**) Time spent in the light box with 200 lux illumination (left) and light/dark transitions (right) during light-dark box test. Unpaired two tailed t test, ns nonsignificant. (**C**) Sucrose preference was tested as the percentage of 1% sucrose intake in all liquid intake within 48 h. Unpaired two tailed t test, ns nonsignificant. (**D**) Immobile time in the forced swimming test. Unpaired two tailed t test, ns nonsignificant. (**E**) Immobile time in the tail suspension test. Unpaired two tailed t test, ns nonsignificant. *n* = 8 mice per group. Data are presented as means ± SEM.


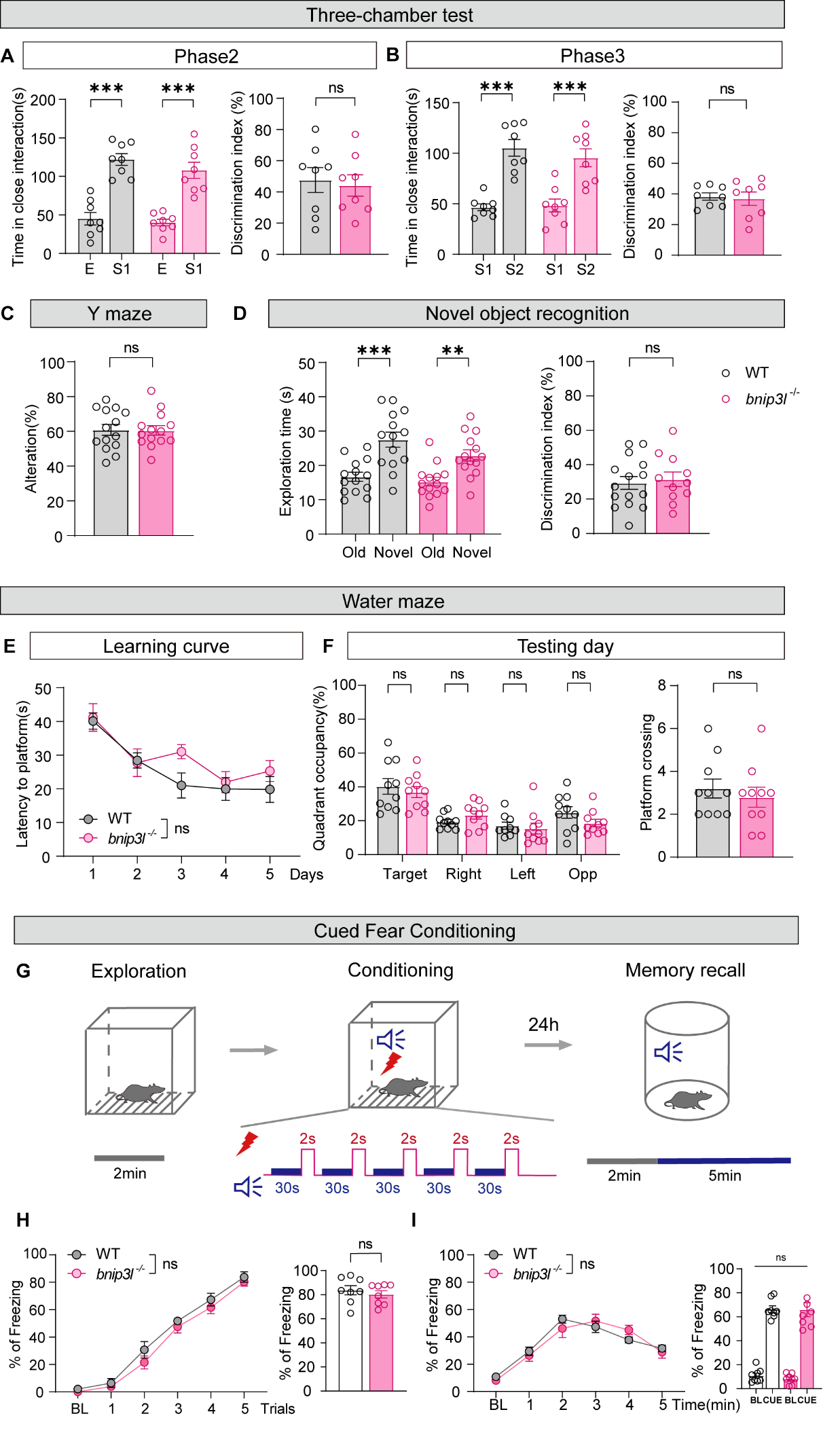


**Figure S4.** The social behavior, learning and spatial memory ability were not altered in *bnip3l^-/-^* mice. (**A**) Time in close interaction with E or S1 (left) and preference index (right) during phase 2. (**B**) Time in close interaction with S1 or S2 (left) and preference index (right) during phase 3. *n* = 8 mice per group. Time in close interaction: paired two tailed t test. Preference index: unpaired two tailed t test, ns nonsignificant. (**C**) Percentage of alteration in the Y maze test. *n* = 10 mice per group. Unpaired two tailed t test, ns nonsignificant. (**D**) Time spent exploring old or novel objects (left) and discrimination index (right) in the novel object recognition test. *n* = 11-15 mice per group. Exploration time: paired two tailed t test, **P* < 0.05. Discrimination index: unpaired two tailed t test, ns nonsignificant. **(E)** Latency to find platform during a five-day navigation trial, two-way repeated measures ANOVA, ns nonsignificant. **(F)** Quadrant occupancy (left) and numbers of platform crossing in a Morris water maze test(right). *n* = 10 mice per group. Unpaired two tailed t test, ns nonsignificant. (**G**) Experimental paradigm. (**H**) The curve of freezing level (left) and the percentage of freezing time (right) on the cued fear conditioning day. The curve of freezing level: two-way repeated measures ANOVA, ns nonsignificant. The percentage of freezing: unpaired two tailed t test, ns nonsignificant. (**I**) The curve of freezing level (left) and the percentage of freezing time (right) on the cued fear recall day. The curve of freezing level: two-way repeated measures ANOVA, ns nonsignificant. The percentage of freezing: unpaired two tailed t test, ns nonsignificant. Unpaired two tailed t test, ns nonsignificant. *n* = 10 mice per group. Data are presented as means ± SEM. **P* < 0.05; ***P* < 0.01; ****P* < 0.001; n.s. *vs.* the indicated group.


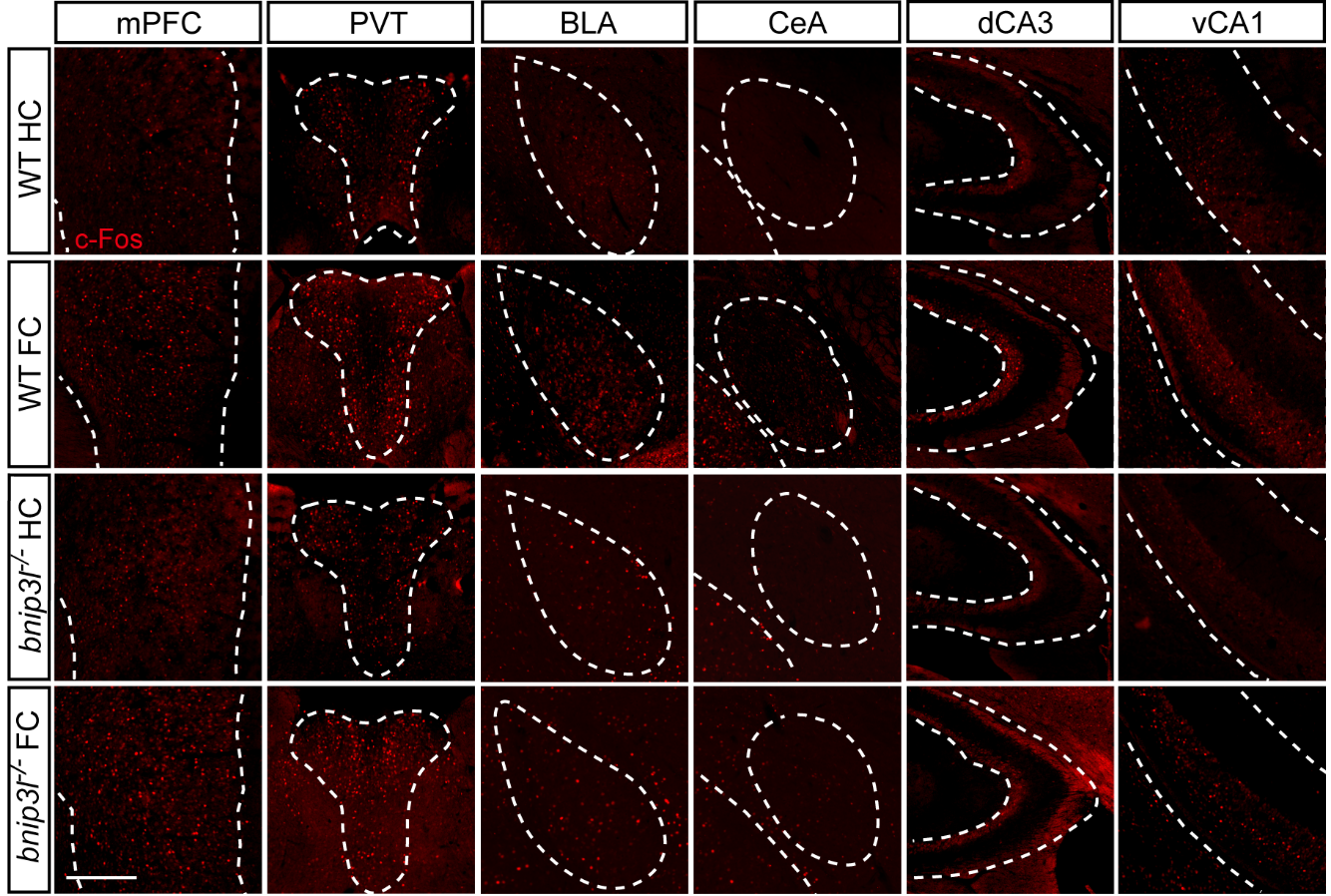
**Figure S5.** Representative images of c-Fos^+^ neurons in regions related with emotion. Scale bar = 200 μm. *n* = 6 mice per group.

**
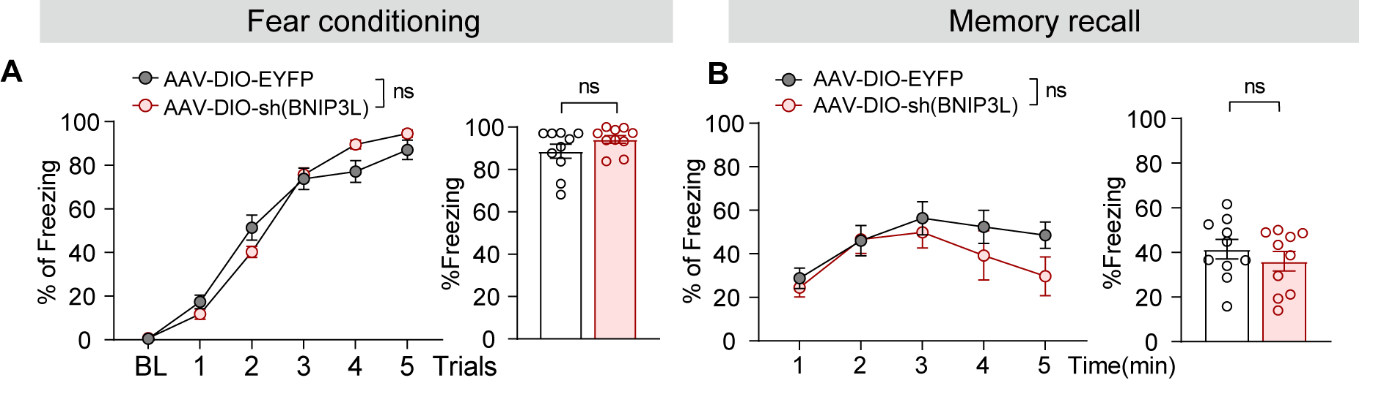
Figure S6.** The vCA1^GLU^-shBNIP3L mice did not impaired fear memory formation. (**A**) The curve of freezing level (left) and the percentage of freezing time (right) on the contextual fear conditioning day. The curve of freezing level: two-way repeated measures ANOVA, ns nonsignificant. The percentage of freezing: unpaired two tailed t test, ns nonsignificant. (**B**) The curve of freezing level (left) and the percentage of freezing time (right) on the contextual fear recall day. *n* = 10 mice per group. The curve of freezing level: two-way repeated measures ANOVA, ns nonsignificant. The percentage of freezing: unpaired two tailed t test, ns nonsignificant. *n* = 10 mice per group. Data are expressed as mean ± SEM.


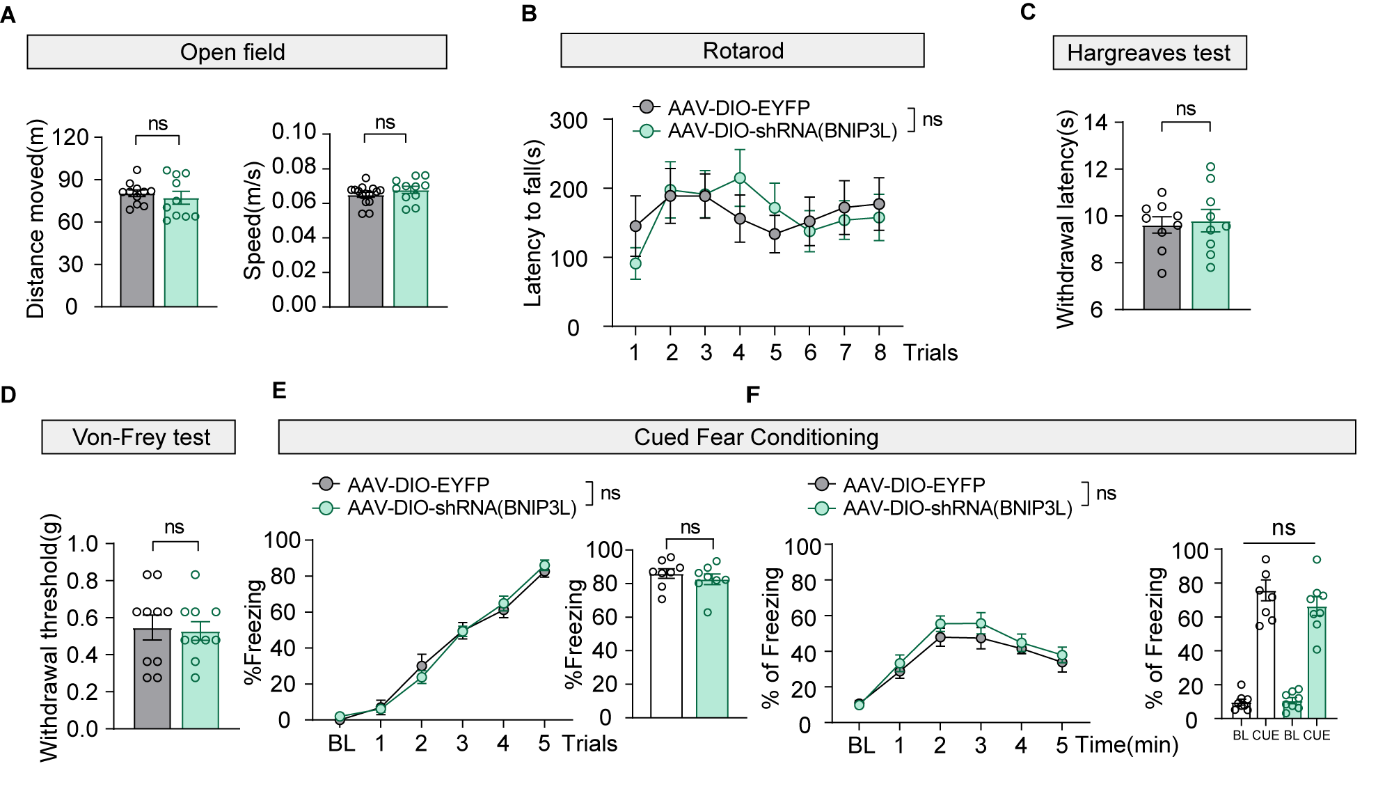
**Figure S7**. The locomotor activity and pain sensitivity were not altered in BLA^GLU^-shBNIP3L mice. (**A**) Total distance moved (left) and speed (right) in the open field over a 30-min period. *n* = 10-11 mice per group. Unpaired two tailed t test, ns nonsignificant. (**B**) Latency to fall in the rotarod test over 8 trials. *n* = 8 mice per group. Two-way repeated measures ANOVA, ns nonsignificant. (**C**) Latency for paw withdrawal in the Hargreaves test. *n* = 9 mice per group. Unpaired two tailed t test, ns nonsignificant. (**D**) Mechanical allodynia paw withdrawal. *n* = 10 mice per group. Unpaired two tailed t test, ns nonsignificant. (**E**) The curve of freezing level (left) and the percentage of freezing time (right) on the cued fear conditioning day. (**F**) The curve of freezing level (left) and the percentage of freezing time (right) on the cued fear recall day. *n* = 8-9 mice per group. The curve of freezing level: two-way repeated measures ANOVA, ns nonsignificant. The percentage of freezing: unpaired two tailed t test, ns nonsignificant. Data are expressed as mean ± SEM.


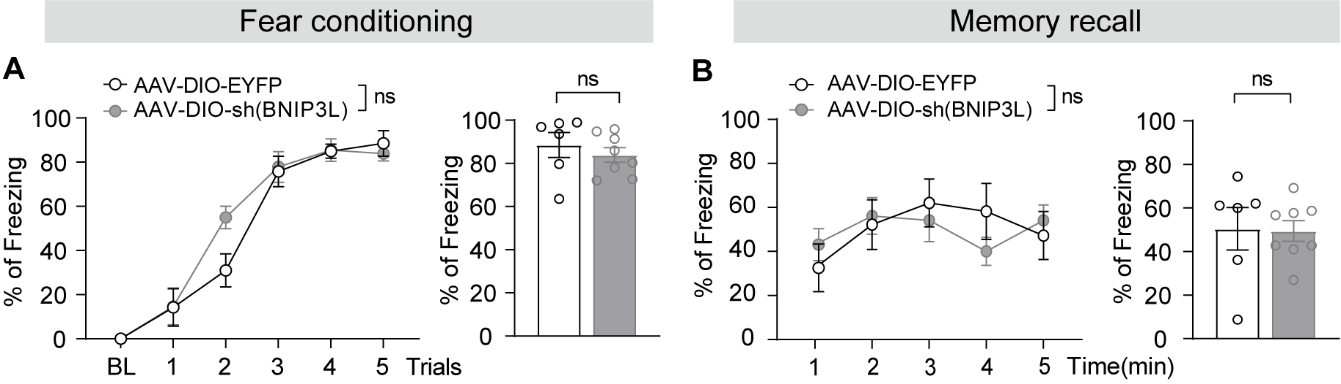
**Figure S8.** The BLA^GABA^-shBNIP3L mice did not impaired fear memory formation. (**A**) The curve of freezing level (left) and the percentage of freezing time (right) on the contextual fear conditioning day. The curve of freezing level: two-way repeated measures ANOVA, ns nonsignificant. The percentage of freezing: unpaired two tailed t test, ns nonsignificant. (**B**) The curve of freezing level (left) and the percentage of freezing time (right) on the contextual fear recall day. The curve of freezing level: two-way repeated measures ANOVA, ns nonsignificant. The percentage of freezing: unpaired two tailed t test, ns nonsignificant. *n* = 6-8 mice per group. Data are expressed as mean ± SEM.

**
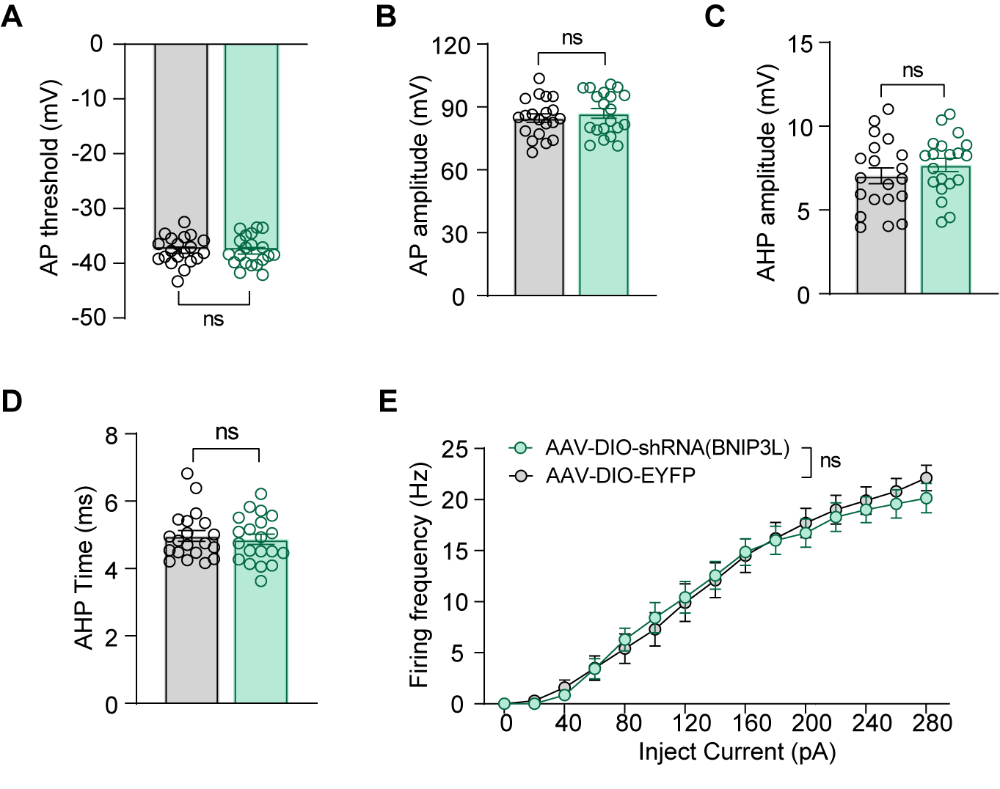
Figure S9.** Deletion of BNIP3L did not change the excitability of BLA glutamatergic neurons. (**A**) Action potential (AP) threshold of BLA glutamatergic neurons. *n* = 20 cells from 4 mice per group. Unpaired two tailed t test, ns nonsignificant. (**B**) AP amplitude of BLA glutamatergic neurons. *n* = 20 cells from 4 mice per group. Unpaired two tailed t test, ns nonsignificant. (**C**) After-hyperpolarization potential (AHP) amplitude. *n* = 20 cells from 4 mice per group. Unpaired two tailed t test, ns nonsignificant. (**D**) After-hyperpolarization potential (AHP) time. *n* = 20 cells from 4 mice per group. Unpaired two tailed t test, ns nonsignificant. (**E**) The firing frequency with depolarizing current injection (from 0 pA to 280 pA, 20 pA steps). *n*=14 cells from 3 mice per group. Two-way repeated measures ANOVA, ns nonsignificant. Data are expressed as mean ± SEM.


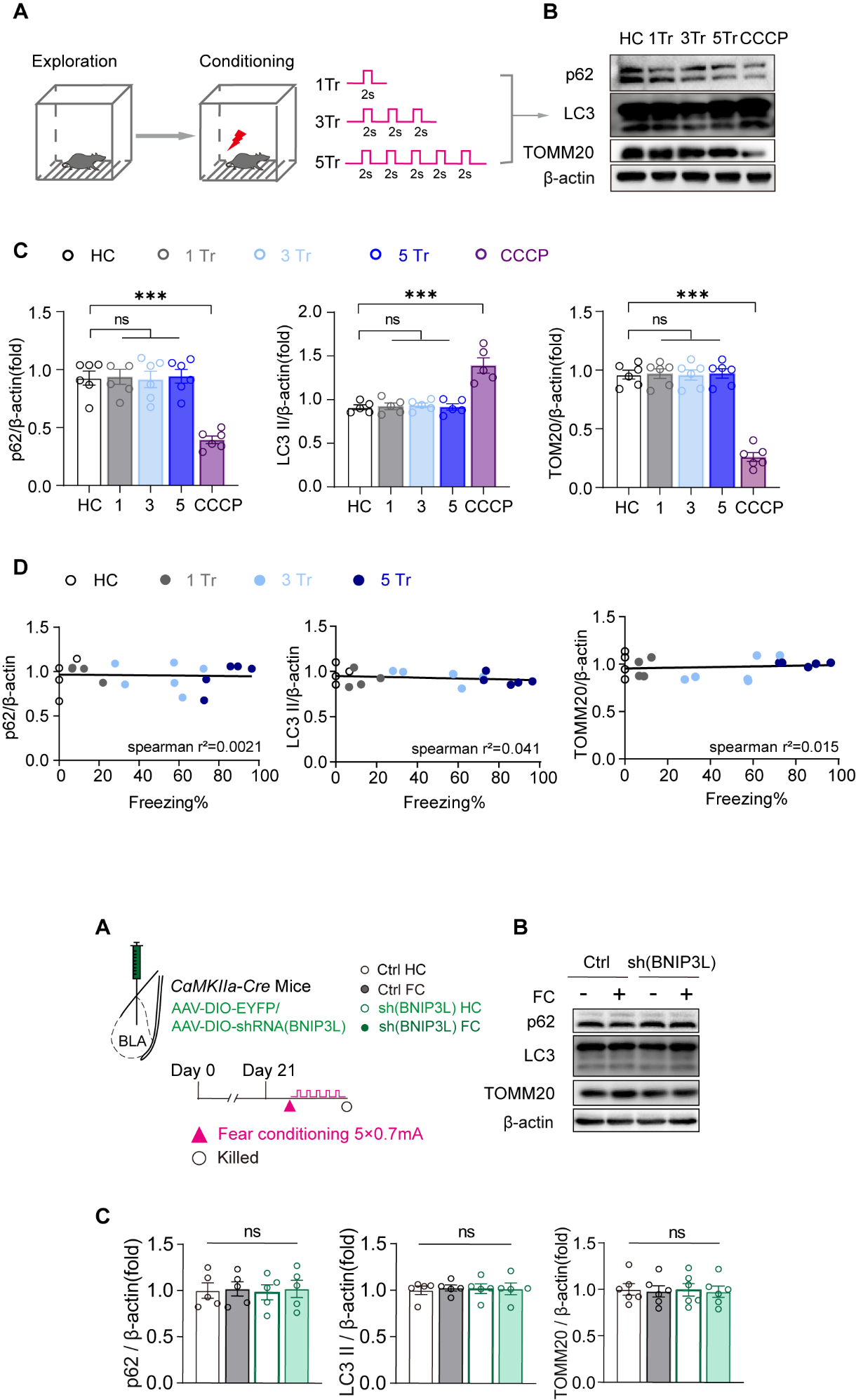
**Figure S10.** Contextual fear conditioning did not reinforce mitophagy in the BLA. (**A**) The two-day contextual fear conditioning test with 0.7 mA foot shock. (**B**) The mice brain tissues were collected. The levels of p62, LC3 and Tom20 were determined by immunoblotting. (**C**) Semi-quantitative analysis of p62, LC3 and Tom20 were shown. (**D**) Analyses of corrections between p62, LC3, Tom20 and freezing level are shown. *n* = 6 mice per group. All experiments are from at least three independent experiments. one-way ANOVA with Tukey’s multiple-comparisons test. **P* < 0.05, ***P* < 0.01, ****P* < 0.001. Data are showed as mean ± SEM. HC, home-cage; Tr, foot-shock trail.


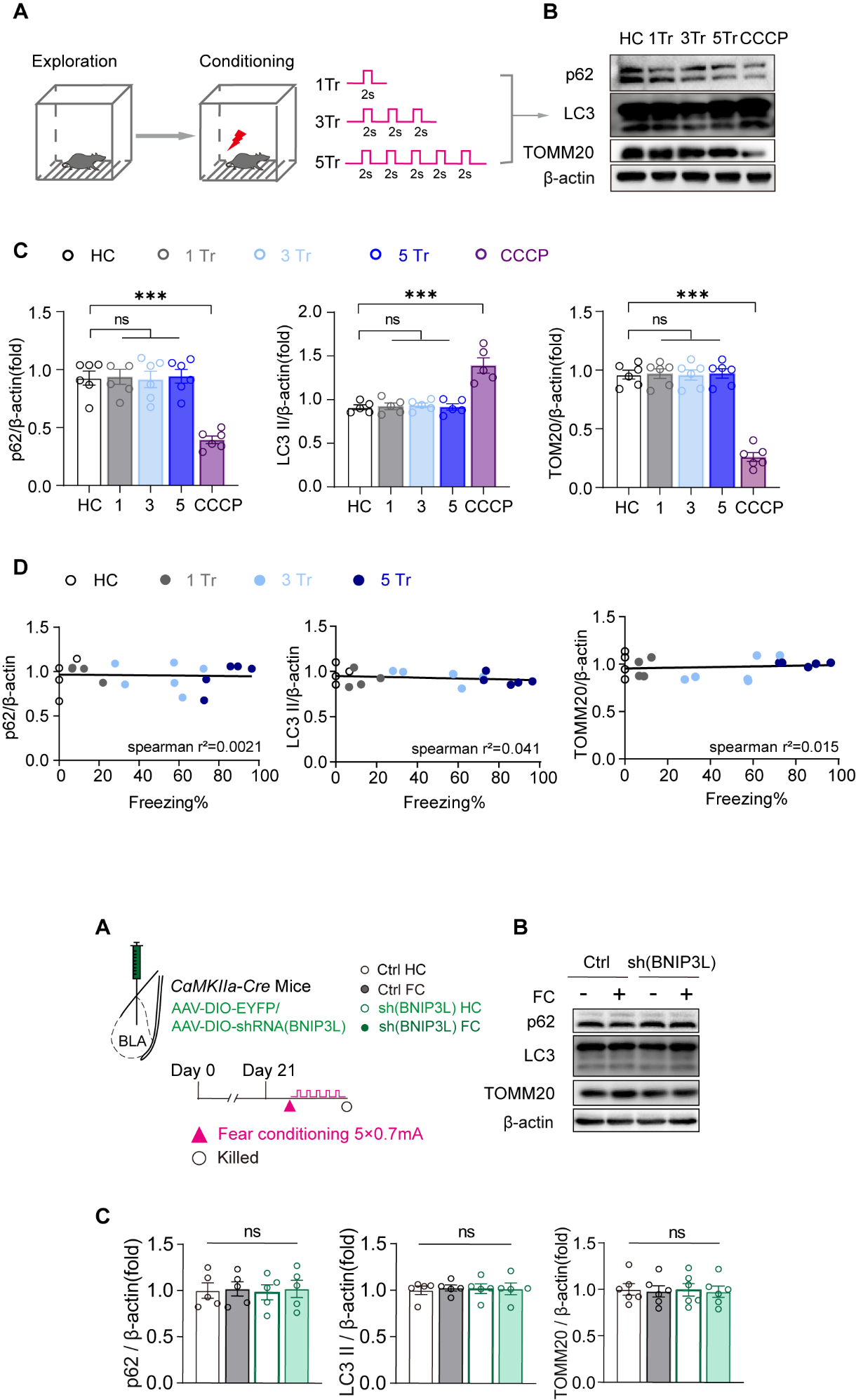
**Figure S11.** Knocking down BNIP3L in the BLA glutamatergic neurons did not reinforce mitophagy during contextual fear conditioning. (**A**) Scheme of AAV injection and fear conditioning. (**B**) The mice brain tissues were collected. The levels of p62, LC3 and Tom20 were determined by immunoblotting. (**C**) Semi-quantitative analysis of p62, LC3 and Tom20 were shown. *n* = 6 mice per group. All experiments are from at least three independent experiments. one-way ANOVA with Tukey’s multiple-comparisons test. n.s., no significance.


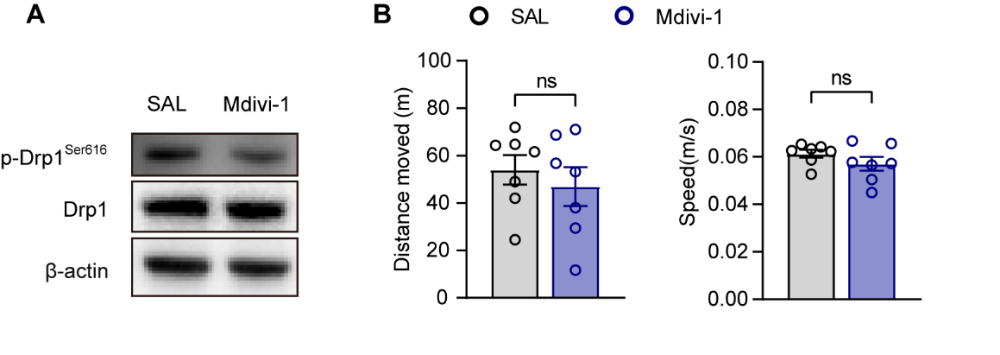
**Figure S12.** Mdivi-1 had no effect on locomotor activity. (**A**) Protein abundance of p-Drp1Ser616 and Drp1 were determined by immunoblotting. (**B**) Total distance moved (left) and speed (right) in the open field over a 30-min period. *n* = 7 mice per group. Unpaired two tailed t test, ns nonsignificant.


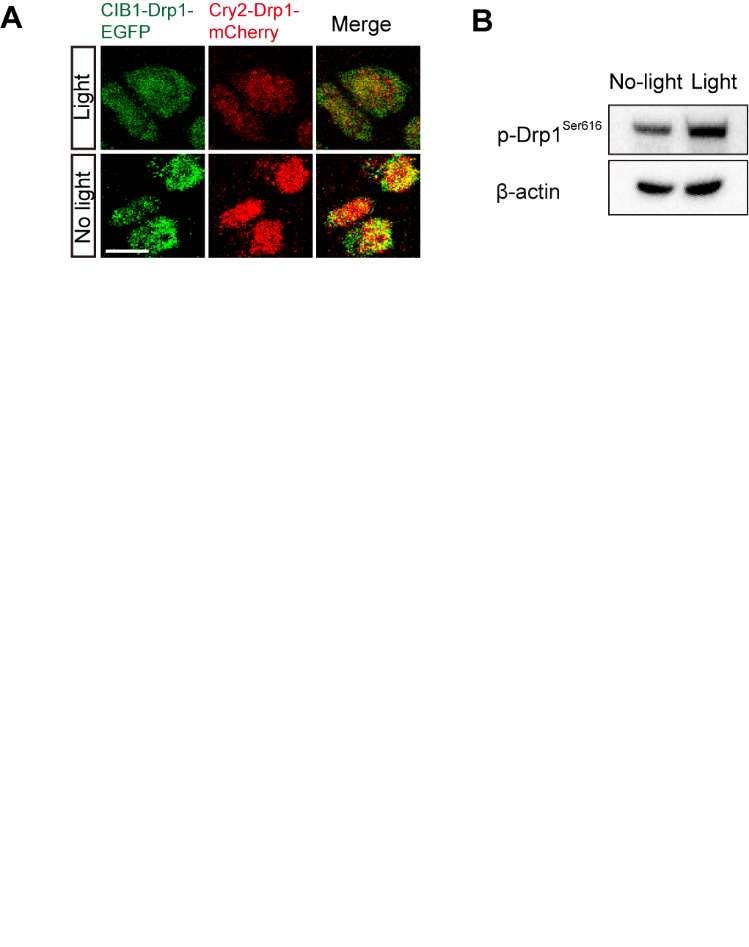


**Figure S13.** Light stimulation induces Drp1 oligomerization and mitochondrial fission. (**A**) Representative images of virus in the BLA; Scale bar = 10 μm. (**B**) Protein abundance of p-Drp1Ser616 and Drp1 were determined by immunoblotting. *n* = 6 mice per group.
